# A CpG island promoter drives the CXXC5 gene expression

**DOI:** 10.1038/s41598-021-95165-6

**Published:** 2021-08-02

**Authors:** Pelin Yaşar, Gizem Kars, Kerim Yavuz, Gamze Ayaz, Çerağ Oğuztüzün, Ecenaz Bilgen, Zeynep Suvacı, Özgül Persil Çetinkol, Tolga Can, Mesut Muyan

**Affiliations:** 1 Department of Biological Sciences, Middle East Technical University, Ankara, 06800 Turkey; 2 Department of Computer Engineering, Bilkent University, Ankara, 06800 Turkey; 3 Department of Chemistry, Middle East Technical University, Ankara, 06800 Turkey; 4 Department of Computer Engineering, Middle East Technical University, Ankara, 06800 Turkey; 5 Cansyl Laboratories, Middle East Technical University, Ankara, 06800 Turkey; 6 Present Address: Epigenetics and Stem Cell Biology Laboratory, Single Cell Dynamics Group, National Institute of Environmental Health Sciences, Research Triangle Park, NC 27709 USA; 7 Present Address: Cancer and Stem Cell Epigenetics Section, Laboratory of Cancer Biology and Genetics, Center for Cancer Research, National Cancer Institute, National Institutes of Health, Bethesda, MD 20892 USA

**Keywords:** Cell biology, Molecular biology

## Abstract

CXXC5 is a member of the zinc-finger CXXC family that binds to unmethylated CpG dinucleotides. CXXC5 modulates gene expressions resulting in diverse cellular events mediated by distinct signaling pathways. However, the mechanism responsible for *CXXC5* expression remains largely unknown. We found here that of the 14 annotated *CXXC5* transcripts with distinct 5′ untranslated regions encoding the same protein, transcript variant 2 with the highest expression level among variants represents the main transcript in cell models. The DNA segment in and at the immediate 5′-sequences of the first exon of variant 2 contains a core promoter within which multiple transcription start sites are present. Residing in a region with high G–C nucleotide content and CpG repeats, the core promoter is unmethylated, deficient in nucleosomes, and associated with active RNA polymerase-II. These findings suggest that a CpG island promoter drives *CXXC5* expression. Promoter pull-down revealed the association of various transcription factors (TFs) and transcription co-regulatory proteins, as well as proteins involved in histone/chromatin, DNA, and RNA processing with the core promoter. Of the TFs, we verified that ELF1 and MAZ contribute to *CXXC5* expression. Moreover, the first exon of variant 2 may contain a G-quadruplex forming region that could modulate *CXXC5* expression.

## Introduction

DNA methylation is one of the mechanisms of gene silencing and primarily occurs in CpG dinucleotides of the genome. Methylation of cytosine residues results in the recruitment of methyl-CpG-binding proteins (MBPs) that act as transcription repressors^1^. Although the majority of CpGs in mammalian genomic DNA is methylated^2^, about 70% of human gene promoters are associated with unmethylated DNA sequences called CpG islands (CGIs)^3,4^. CGIs, which are rich in C and G nucleotides and defined by a high density of CpG dinucleotides, are often refractory to methylation and characterized with a chromatin state permissive for transcription. Recent studies indicate that deregulation of the tissue-specific methylation state of different classes of CGI promoters could contribute to the initiation/progression of cancer^5,6^. The establishment and maintenance of CGI-specific chromatin conditions are mediated by structurally and functionally distinct zinc-finger (ZF)-CXXC family proteins. The ZF-CXXC proteins preferentially interact with unmethylated CpG dinucleotides through a highly conserved ZF-CXXC domain characterized by two consecutive cysteine-rich motifs (CXXCXXC) tetrahedrally coordinated with Zn^2+^ ions forming zinc-finger structures. Upon binding to DNA, the ZF-CXXC proteins establish a chromatin architecture directly through chromatin-modifying enzymatic activities and/or indirectly through the recruitment of chromatin-modifiers^7–9^.

CXXC5, also known as RINF and WID, is a member of the ZF-CXXC family^8,10^. The CXXC5 gene located on chromosome 5q31.2 is ubiquitously expressed, albeit at varying levels, in human tissues^8,10^. Evidence indicates that morphogenic retinoic acid^11^, multifunctional cytokine family member transforming growth factor-β^12^, bone morphogenetic protein 4^13,14^, the Wnt family of secreted glycolipoprotein Wnt3a^15–17^ or estrogen^18–20^ alters the *CXXC5* expression as the primary response gene, whose protein product subsequently leads to changes in cell type-specific secondary gene expressions^13,17,21–26^. These changes are manifested as the modulation of cellular metabolism, proliferation, differentiation, or death in developmental processes and tissue maintenance^11–13,15,21,23,24,26–31^. Consistent with the functional importance of CXXC5 in physiology, de-regulated expressions of *CXXC5* have been reported to correlate with the development of various pathologies including acute myeloid leukemia (AML), gastric, prostate, and breast cancer^11,32–38^.

Despite the involvement of CXXC5 in diverse cellular events mediated by distinct signaling pathways, the mechanism responsible for the expression of the CXXC5 gene remains largely unknown. Spatio-temporal control of gene expression is achieved at the transcriptional level. This requires the integrated effects of sequence-specific trans-factors, general transcription regulators, and cis-acting DNA regulatory elements including promoters, promoter-proximal elements, distance-independent elements, locus control regions, and insulator within a highly dynamic chromatin environment^39,40^. Nevertheless, promoters as diverse and complex architectural DNA segments primarily located adjacent to the transcriptional start sites (TSSs) of genes constitute the key platform for the assembly of pre-initiation complexes to mediate transcription^39,40^. Delineation of promoter features of the CXXC5 gene is essential for understanding the mechanisms of the CXXC5 gene regulation in a signal pathway- and cell type-dependent manner that could underlie its role in physiology and pathophysiology.

We found here that of the 14 annotated *CXXC5* transcripts, transcript variant 2, which is composed of Exon3, 10, and 11 of *CXXC5* and has the highest expression level among transcript variants, represents the main *CXXC5* transcript in cell models. We also identified a DNA segment in and at the 5′ sequences of the first exon of transcript variant 2 (Exon3) as the core promoter region. Based on DNA sequence composition and motifs, chromatin configuration of as well as the presence of multiple TSSs together with an active RNA polymerase II at the core promoter, we suggest that a CGI promoter drives the expression of *CXXC5*. A promoter pull-down approach revealed the potential association of various transcription factors (TFs)/co-regulatory proteins, as well as of proteins involved in histone/chromatin, DNA, and RNA processing with the core promoter. We found here that of the transcription factors, ELF1 and MAZ contribute to the *CXXC5* expression. Moreover, the DNA sequence within the first exon of transcript variant 2 was found to form a G-quadruplex (G4) structure in vitro that could modulate the *CXXC5* expression *in cellula*.

## Results

**Figure 1:**
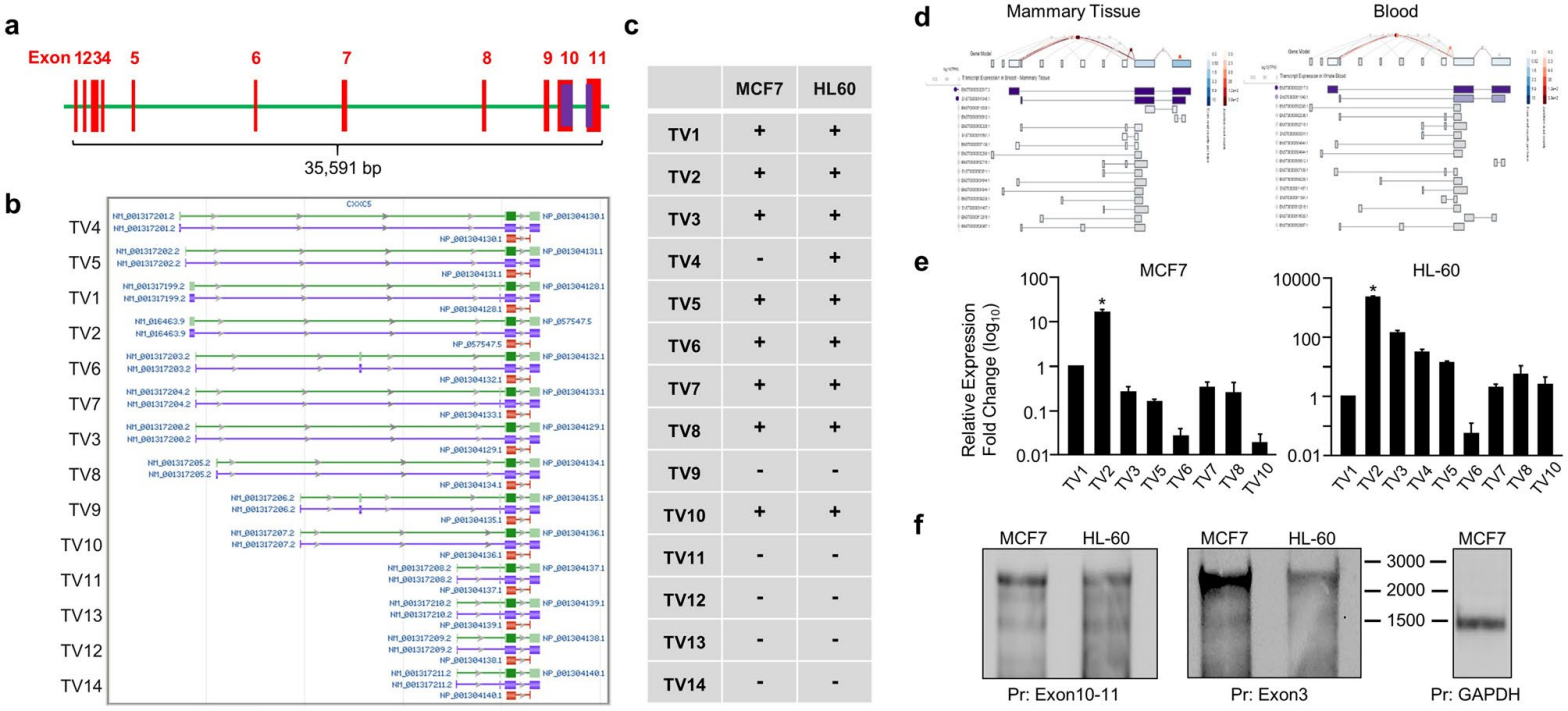
Expression of transcription variants (TVs) in MCF7 and HL60 cells. (**a**) The *CXXC5* gene contains 11 exons and 10 introns. (**b**) A screenshot of transcript annotations by Genome Data Viewer of NCBI indicates the presence of 14 transcript variants encoded by the *CXXC5* gene. (**c**) The presence of CXXC5-transcript variants in MCF7 and HL60 cells was assessed with multiple rounds of PCRs with progressively nested primers specific to a variant together with a primer specific to Exon10, which is common to all CXXC5-transcript variants, followed by cloning and sequencing of the PCR amplicons. (**d**) Screenshots of the expression levels of CXXC5-transcript variants in healthy breast tissue and blood annotated by GTEx Portal. (**e**) qPCR of cDNA libraries was used to assess the expression levels of transcript variants in MCF7 and HL60 cells using primer sets also utilized in evaluating the presence of transcript variants. Asterisk (*) indicates the highest expression among transcript variants. (**f**) Northern blot analyses of RNA samples from MCF7 or HL60 cells. A biotinylated probe complementary to Exon10/11 present in all transcript variants and a biotinylated probe targeting Exon3 were used for the detection of the *CXXC5* transcripts. A *GAPDH* targeting probe was also used for the detection of the *GAPDH* transcripts as control. The molecular ladder (nt) is indicated.

Genes can have multiple transcript variants encoding the same protein or different variants of a protein, and/or non-coding transcript variants as a result of alternative promoter usage and/or alternative splicing^41–43^. Alternative splicing of a single gene can generate a repertoire of protein isoforms with distinct features. Alternative promoters of a gene could have different tissue specificity, developmental activity, expression levels, and they may produce protein isoforms with distinct amino-termini^41–43^. The CXXC5 gene located on the long arm of chromosome 5, in the 5q31.2 region, is approximately 35 kb in length according to the human genome assembly GRCh38. The gene contains 11 exons and 10 introns. Transcript annotations by databases including Ensembl (https://www.ensembl.org/index.html), NCBI (https://www.ncbi.nlm.nih.gov) indicate that the CXXC5 gene can generate 14 transcript variants in different tissues (Fig. 1a,b), identified by the Expressed Sequence Tags (ESTs) approach widely utilized to identify alternative splicing products of genes^44,45^. The last two exons of the gene are found in all 14 transcript variants and contain the coding region of 969 nucleotides (nt), 924 bp of which is in Exons10, and 45 bp including a stop codon is in Exon11. All 14 transcript variants of *CXXC5* encode the same protein with a calculated molecular mass (MM) of 33 kDa.

Since annotation of promoters in the human genome relies on the experimental evidence of 5′-ends of mRNA transcripts, which primarily correspond to the transcription start sites^46,47^, we predicted that the identification of TSS(s) of the main transcript variant(s) as the quantitatively most abundant one(s) could be used to define the promoter region(s) of *CXXC5*. The expression of *CXXC5* as a retinoid-inducible nuclear factor critical for retinoid-induced cellular differentiation was first reported in cell models, including HL60 cells, derived from leukemia^11,48^. Similarly, we showed using breast adenocarcinoma-derived cell lines, including MCF7 cells, that CXXC5 is an E2-ER responsive gene^18,19^ and CXXC5 as an unmethylated CpG binder contributes to E2-mediated gene expressions critical for cellular proliferation^29^. We, therefore, explored the presence and the extent of transcript variant expressions of *CXXC5* in MCF7 cells and HL60 cells as cell models. To identify transcript variants of CXXC5 expressed in MCF7 and HL60 cells, cDNA libraries generated from total RNA were subjected to multiple nested rounds of PCR using progressively nested primers specific to each variant together with a primer specific to Exon10, which is common to all *CXXC5* transcript variants, followed by cloning and sequencing of the PCR amplicons (Supplementary Information, Table 1). Results revealed that *CXXC5* generates transcript variants 1–3, 5–8, and 10 in MCF7 cells; whereas transcript variants 1–8 and 10 were detectable in HL60 cells (Fig. 1c). These findings suggest that the *CXXC5* gene, except for transcript variant 4 which is present in only HL60 cells, generates the same transcript variants in both cell lines.

Our GTEx Portal analyses (www.gtexportal.org) indicate that although transcript variants of *CXXC5* are expressed at varying levels in distinct tissues, transcript variant 2 and transcript variant 3 in breast tissue and transcript variant 2 in blood show the highest expression levels (Fig. 1d). Consistent with these, our qPCR results with the cDNA libraries used in the detection of transcript variants revealed that CXXC5-transcript variant 2 (NCBI Accession: NM_016463, Version: NM_016463.9), which contains Exons 3, 10, and 11 is the transcript with the highest relative expression in both MCF7 and HL60 cells (Fig. 1e).

Based on these results, we carried out Northern Blot (NB) analyses using ribosomal-RNA depleted RNA samples from MCF7 or HL60 cells. The length of the annotated human *CXXC5* transcript variants varies between 2163 and 2695 (retrieved on March 2021, https://www.ncbi.nlm.nih.gov/datasets/tables/genes/?table_type=transcripts&key=37c49fee33e03d725b813c4cda693206) nt. We used a biotinylated probe complementary to the joint boundaries of Exon10 and Exon11 (343 nt in length) which are present in all transcript variants, and a biotinylated probe targeting Exon3 sequences (390 nt). We also used a *GAPDH* probe (589 nt) targeting Exon5-8 to detect the *GAPDH* transcript of 1525 nt in length as the control (NCBI Accession: NM_001289746, Version: NM_001289746.2). Results revealed that both probes specific to *CXXC5* detect primarily a CXXC5 transcript with an electrophoretic migration of approximately 2500 nt in length similar to that of the annotated transcript variant 2, 2601 nt, while, as expected, a single *GAPDH* transcript of about 1500 nt was detected (Fig. 1f).

Because of these findings, we predicted that the promoter of *CXXC5* resides in a transcript variant 2 region that encompasses a transcription start site (TSS). For the identification of TSS(s), we used the 5’ Rapid Amplification of cDNA Ends (5’RACE) approach, designed for the amplification of nucleic acid sequences from a messenger RNA (mRNA) template between a defined internal site and unknown sequences at the 5′-end of the mRNA through the use of an adaptor RNA probe^49^. Although prone to biases introduced by various factors including RNA secondary structures, G–C nucleotide content, adaptor ligation efficiency^50^, 5′RACE has been successfully used for the identification of 5′-ends of numerous RNA transcripts^51^. We also used *TFF1*, a well-studied estrogen-responsive gene^19,52^, as a control for 5′RACE studies. 3′RACE was also used for the identification of 3′-transcript sequences of both *CXXC5* and *TFF1* transcripts.

**Figure 2:**
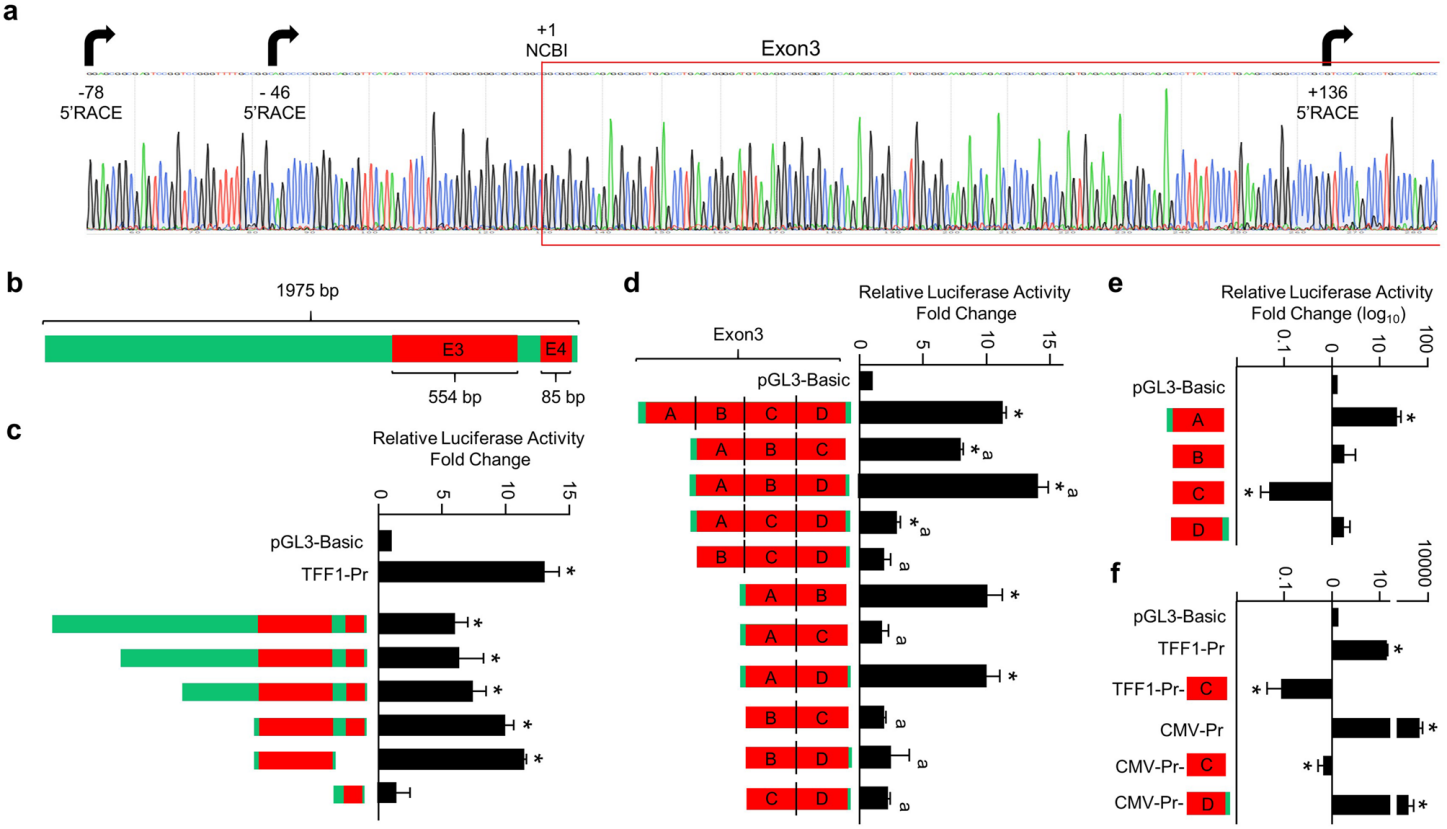
Assessing the putative promoter region of *CXXC5*. (**a**) The chromatogram of the longest sequence of transcript variant 2 identified by the 5’RACE approach is shown. Arrows indicate transcription start sites obtained with 5’RACE, + 1 denotes the start of Exon3 according to NCBI (Reference Sequence: NM_016463.9). (**b**) Schematics of a *CXXC5* region encompassing 3’ end of Intron2, Exon3, and Exon4 as the full-length template for reporter assays. (**c–f**) MCF7 cells grown in growth medium for 48 h with 10% FBS were transiently transfected with pGL3 bearing none (Basic-Luc), the promoter of estrogen-responsive *TFF1* gene, or the template *CXXC5* region bearing the full-length, or a 5′- and/or 3′end truncated *CXXC5* region that drives the Firefly *Luciferase* cDNA expression as the reporter enzyme for 24 h. The transfection efficiency was monitored by the co-expression of pCMV-RL that drives the expression of the *Renilla Luciferase* cDNA. Cellular extracts were then subjected to dual-luciferase assays. (**d**, **e**) Exon3 with four segments (A-D) was cloned as the full-length, various combinations of segments or a segment alone into the Basic-Luc reporter vector. (**f**) Segment C or Segment D of Exon3 was genetically fused to the 3′-end of the *TFF1* or CMV promoter driving the expression of the Firefly *Luciferase* cDNA as the reporter enzyme. In all reporter assays, conducted in transiently transfected MCF7 cells, shown are the mean ± SE of three independent experiments performed in triplicate. Firefly/*Renilla* luciferase activities are presented as fold change compared to pGL3-Basic, which is set to one. Asterisk (*) indicates significant differences from the Basic-Luc control. The superscript “a” indicates a significant difference compared to Exon3.

The 3’RACE approach readily identified the 3′ end of the *CXXC5* or *TFF1* transcript (Supplementary Information, Fig. S1). 5’RACE of *CXXC5*, in contrast to that of *TFF1* which generates a transcript with a single TSS^53^ (Supplementary Information, Fig. S1), proved to be difficult likely due to the high GC content (> 70%) of the Exon 3 and surrounding sequences. Nevertheless, our results based on the sequencing of PCR amplicons generated from cDNA libraries of MCF7 cells indicated that several 5′-ends of transcript variant 2 can be detected, suggestive of multiple TSSs (Fig. 2a). These results imply the presence of a transcription start region for transcript variant 2 rather than a distinct TSS.

Collectively, our results indicate that transcript variant 2 of *CXXC5* composed of Exons 3, 10, and 11 is the main transcript in MCF7 and HL60 cells.

### The *CXXC5* promoter is located in a DNA segment encompassing the beginning of Exon3

Based on the similar results obtained in MCF7 and HL60 cells, we assessed the promoter activity of the putative promoter region of *CXXC5* by generating a PCR amplicon of 1975 bp in length from MCF7 genomic DNA that includes 5′ upstream regions of Exon3, the entire Exon3, and Exon4 (Fig. 2b). The PCR amplicon was inserted into a reporter vector, pGL3-Basic, which has no promoter but bears the Firefly Luciferase cDNA as the reporter enzyme. We also used a reporter vector bearing the estrogen-responsive *TFF1* gene promoter^19^ as control. We found in transiently transfected MCF7 cells that the reporter enzyme activity from the putative *CXXC5* promoter region, as from the *TFF1* promoter, was significantly higher compared to that observed with the reporter vector bearing no promoter, from which the enzyme activity is set to one (Fig. 2c). To decipher the core promoter elements of the putative *CXXC5* promoter, we generated sequential truncations at the 5′- or 3′-end of the region by PCR and inserted them into the reporter vector. Results from transiently transfected MCF7 cells indicated that DNA sequences of Exon3 produce the highest reporter activity (Fig. 2c). Further truncations and/or internal deletions as Segments (A–D) of Exon3 revealed that Segment A, corresponding to, and including the 5′ surrounding sequences of, Exon3 (Supplementary Information, Fig. S2) retains the promoter activity (Fig. 2d,e). Interestingly, Segment C alone suppresses (Fig. 2e), and in the presence of other segments lessens (Fig. 2d) the activity of the reporter enzyme. This suggests that Segment C alone includes DNA elements adversely affecting transcription. In keeping with this prediction, the genetic fusion of Segment C, to the 3′-end sequences of the *TFF1* promoter or of the strong human cytomegalovirus (CMV) promoter effectively repressed the *Luciferase* enzyme activity (Fig. 2f) in contrast to Segment D which has minimal effects on the reporter activity induced by the CMV promoter.

These results suggest that the core promoter elements of *CXXC5* reside in Segment A.

### The methylation state of the putative *CXXC5* promoter region

**Figure 3:**
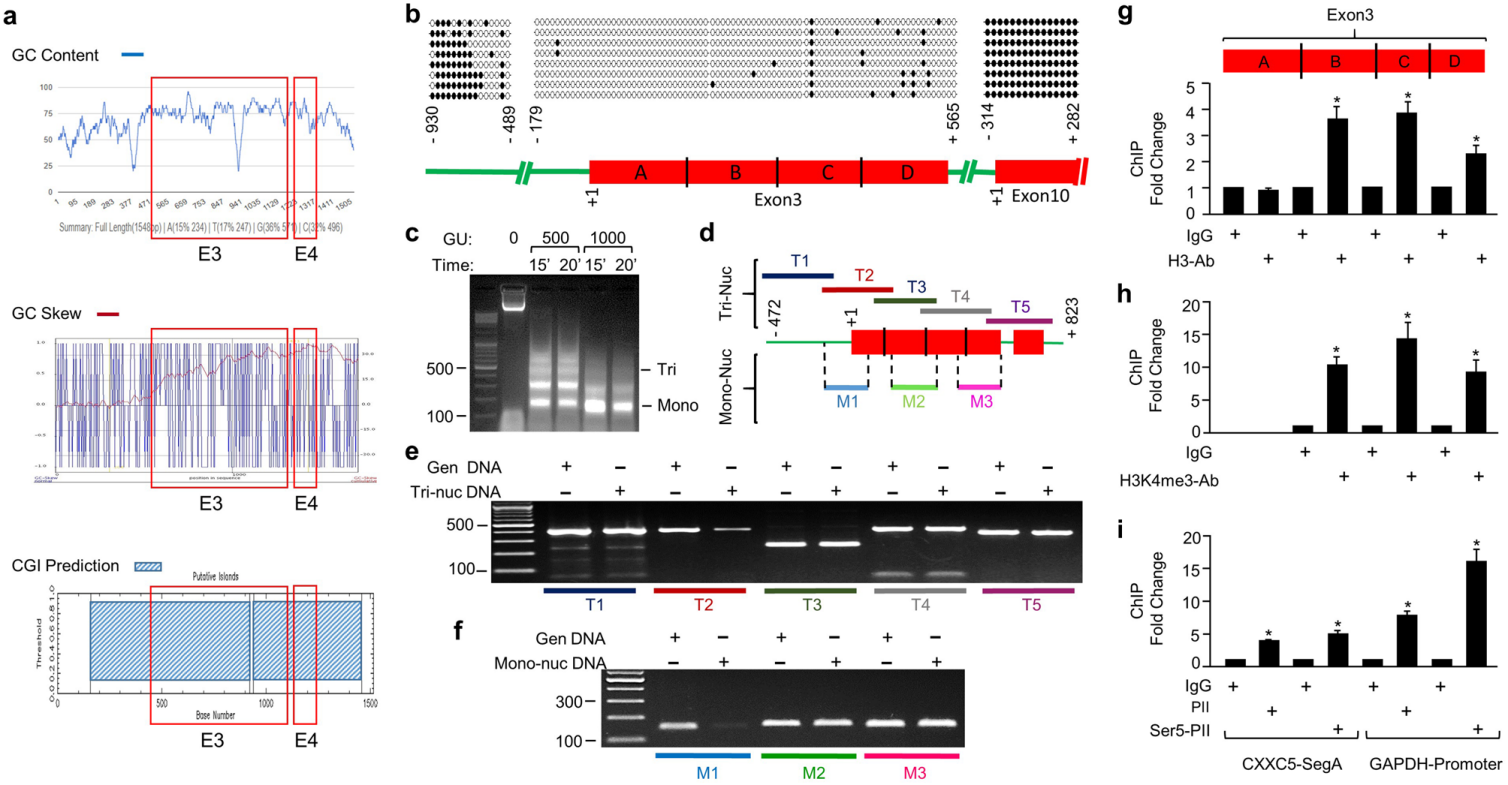
Feature of the *CXXC5* region encompassing the promoter of *CXXC5*. (**a**) In silico analyses of the nucleotide sequence of the region for G-C content, asymmetric GC distribution, GC skew (GC skew), and the presence of a CpG island (CGI). (**b**) The methylation state of the *CXXC5* promoter region (-930 to -489 and -179 through the Exon3; + 1 indicates the beginning of Exon3) together with the 3’-end of Intron9 and Exon10 (− 195 through + 330; + 1 marks the beginning of Exon10) as controls was examined with bisulfite sequencing. Isolated genomic DNA of MCF7 cells was subjected to bisulfite reaction for the conversion of unmethylated cytosine residues to uracil followed by bisulfite PCR. PCR amplicons produced with bisulfite primers were cloned and sequenced. Aligned sequences to the corresponding *CXXC5* regions were depicted as a lollipop distribution. Filled circles indicate methylated and empty circles denote unmethylated CpG dinucleotides. (**c**) Nucleosome occupancy at the *CXXC5* promoter elements was assessed with Micrococcal Nuclease (MNase) assay. MCF7 cells were fixed, permeabilized, and treated without (0) or with 500 or 1000 gel units (GU) of MNase for 15 or 20 min at 37 °C for chromatin digestion. Isolated DNA was analyzed with agarose gel electrophoresis. (**d**) Schematics of regions subjected to PCR. Isolated DNA fragments corresponding to (**e**) tri-nucleosomal (T1-5) and (**f**) mono-nucleosomal (M1-3) DNA were subjected to PCR using region-specific primer pairs. (**g**–**i**) ChIP analysis of Exon3. Following chromatin digestion of MCF7 cells by the use of MNase were subjected to ChIP using species-specific IgG, an antibody specific to total H3 (**g**) or H3K4me3 (**h**). Isolated DNA following precipitation with Protein A/G conjugated magnetic beads was subjected to qPCR using region-specific primer sets. Asterisk (*) denotes significant changes depicted as fold change compared to IgG. (**i**) MCF7 cells fixed, lysed, and sonicated were subjected to ChIP using IgG, PolII, or ser5-phosphorylated PolII antibody followed by precipitation with Protein A/G conjugated magnetic beads. DNA samples were then used for qPCR with primer sets specific to Segment A or the promoter of *GAPDH* as control. Shown are the mean ± SE of three independent experiments performed in triplicate. Significant differences depicted with an asterisk (*) are shown as fold change compared to IgG.

Based on the conclusion that transcript variant 2 is the main *CXXC5* transcript in both MCF7 and HL60 cells, we initially carried out in silico analyses of a genomic region, about 1500 bp in length, of the *CXXC5* locus, wherein Exon3 is situated, as the putative promoter region (Fig. 3a). The nucleotide sequence of the region revealed (1) a remarkably high (> 70%) G–C content (https://www.biologicscorp.com/tools/GCContent/), (2) a greatly enriched CpG dinucleotide repeats (https://www.biologicscorp.com/tools/GCContent/), (3) an asymmetric GC distribution, GC skew, which is used as a measure of DNA strand asymmetry in the GC nucleotide distribution (http://genskew.csb.univie.ac.at/GenSkewServlet) as a property of CpG islands^54^, and (4) the presence of a CpG island (CGI) (EMBOSS Cpgplot; https://www.ebi.ac.uk/). These analyses suggest that the transcription start region, including Segment A, of the transcript variant 2 is located in a CpG island, a conclusion consistent with the CGI annotation track of the *CXXC5* locus in the human genome (https://genome.ucsc.edu/).

The methylation of mammalian genomic DNA shows variations across cell types, developmental stages, physiological and pathophysiological conditions^55^. Acting as stable and heritable epigenetic marks, methylated CpGs are present in 80% of CpGs in the genome and involve both genic and intergenic regions^2^. Although the majority of CpGs are methylated, about 70% of human gene promoters are associated with unmethylated CGIs^3,4^. CGIs are short (200–2000 bp) DNA segments that display high G-C content with enriched CpG dinucleotide repeats^3,4,56^. CGI promoters, often define promoters of housekeeping, developmental and tissue‐specific genes, show a transcriptionally permissive state, within which transcription initiation can occur at several closely spaced locations^3,4,56^. To examine the methylation state of Exon3 and the surrounding region including the putative *CXXC5* promoter segment, we explored a targeted methylation profile of the region as well as Exon10 as a control for the methylated gene body of the *CXXC5* locus using bisulfite-sequencing. Genomic DNA of MCF7 cells was subjected to bisulfite reaction to convert unmethylated cytosine residues to uracil followed by bisulfite PCR. PCR amplicons generated with bisulfite primers were cloned and sequenced. Sequences were then aligned to the genomic sequence of the corresponding *CXXC5* regions using QUMA^57^ (http://quma.cdb.riken.jp/). Results from MCF7 (Fig. 3b) as well as HL60 cells (Supplementary Information Fig. S3) indicated that the 5′-upstream region of Exon3 shows a high degree of CpG methylation, which declines precipitously thereafter and remains largely unmethylated throughout the region including Exon3 (Fig. 3b) and Exon4 (data not shown). This contrasts with Exon10 which is highly methylated (Fig. 3b).

Common with all eukaryotic promoters, unmethylated CGI promoters also possess a nucleosome-free region surrounding TSSs^58^ and contain dispersed nucleosomes decorated with H3K4me3, which marks active transcription^59–61^. To assess the nucleosome occupancy at the DNA region including the putative *CXXC5* promoter elements, MCF7 cells were fixed, permeabilized, and subjected to Micrococcal Nuclease (MNase) for chromatin digestion (Fig. 3c). DNA was subsequently purified and analyzed for digestion patterns with agarose gel electrophoresis. DNA fragments corresponding to tri-nucleosomal and mono-nucleosomal DNA were excised from the gel and purified. The fragmented DNA, or the uncut genomic DNA of MCF7 cells as control, was used as the template for PCR to assess the presence of nucleosomes at Exon3. For initial analyses, five overlapping regions (depicted as T1-5, Fig. 3d) were subjected to PCR using the tri-nucleosomal DNA template with the region-specific primer pairs. For further verification, three sub-regions of Exon3 (depicted as M1-3, Fig. 3d) were also analyzed using the mono-nucleosomal DNA. The detection of a PCR amplicon from fragmented DNA compared to genomic DNA suggests the presence of nucleosomes. Results with tri- (Fig. 3e) or mono-nucleosomal (Fig. 3f) DNA template revealed that the 5′-surrounding sequences of Exon3 and Segment A are primarily nucleosome-deficient and the remaining segments of Exon3 contain nucleosomes. To verify this finding, we carried out ChIP of Exon3 (Fig. 3g,h). MCF7 cells processed for chromatin digestion by the use of MNase, as described for nucleosome occupancy, were subjected to ChIP using an antibody specific to H3 (Fig. 3g) or tri-methylated histone H3 lysine 4, H3K4me3, (Fig. 3h), a histone modification used as a marker for actively transcribed genes^60^. Purified DNA was then subjected to qPCR using primers specific to Segments of Exon3. We found that Segment A is indeed devoid of H3 but the remaining segments of Exon3 bear H3 decorated with K4me3 modification. We also observed the presence of an active PolII at Exon3, shown on Segment A, as on the promoter of the housekeeping *GAPDH* gene as control, using ChIP-qPCR with an antibody specific to PolII or Ser5 phosphorylated PolII (Fig. 3i).

Our results collectively indicate that Segment A of Exon3 constitutes the core promoter element of *CXXC5* located in a CGI.

### Identification of proteins engaged with the *CXXC5* core promoter

**Figure 4:**
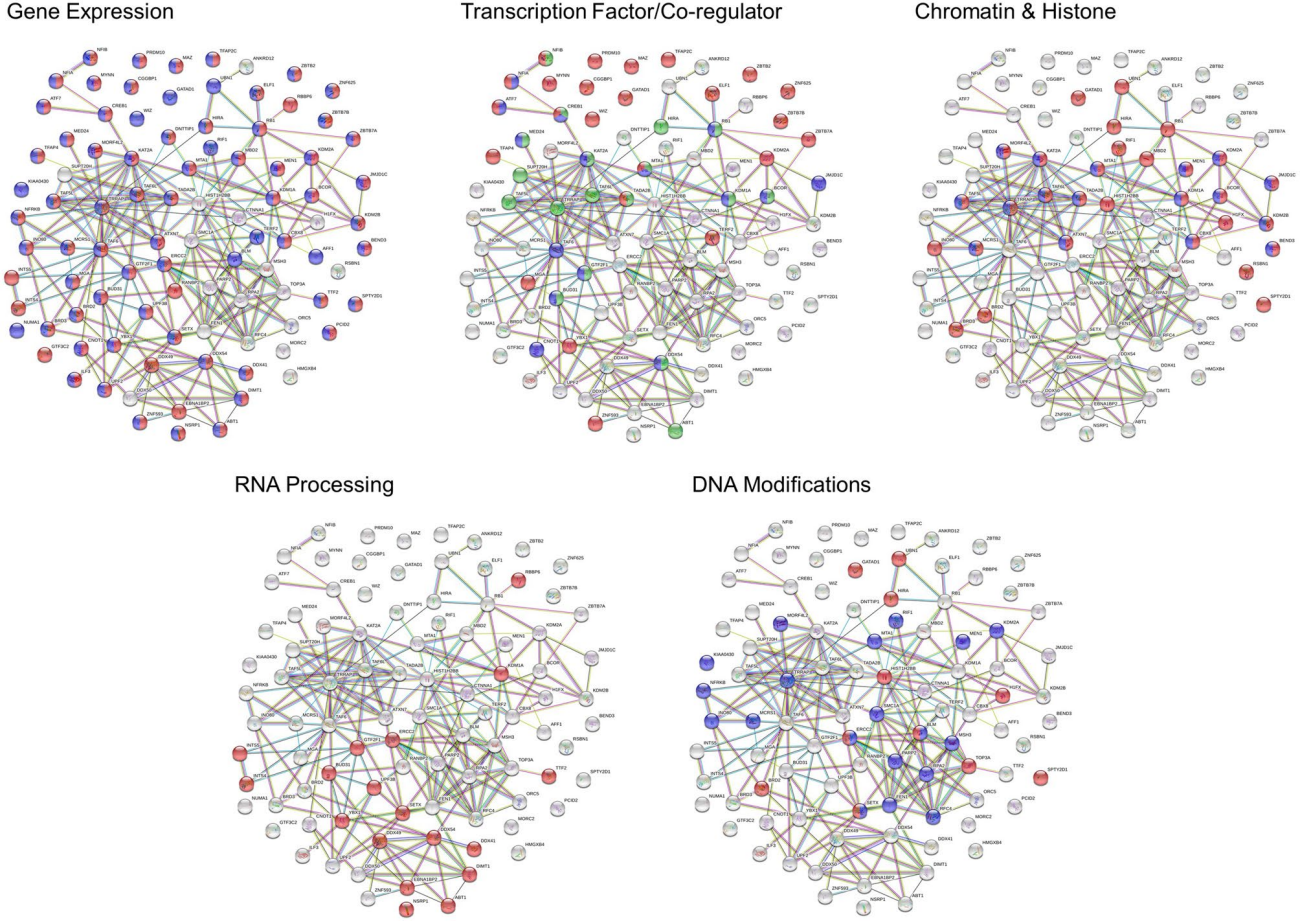
GO term enrichment analyses of proteins associated with Segment A as the core *CXXC5* promoter element using the STRING interaction database. Nuclear extracts of MCF7 cells were incubated overnight with a 5′-end biotinylated PCR amplicon containing Segment A or a fragment of Exon10 as the control followed by precipitation with streptavidin-conjugated magnetic beads. Bound proteins were then subjected to MS for protein IDs. Proteins are grouped under the headings of Gene Expression [GO: 0010467 Regulation of gene expression (red) and GO: 0010468, Gene expression (Blue)]; Transcription Factor/co-Regulator [GO: 0000981, DNA-binding transcription factor activity (red), GO:0008134, Transcription factor binding (blue), and GO: 0003712, Transcription co-regulator activity (green)]; Chromatin and Histone [GO: 0006325, Chromatin organization (red) and GO: 0016570, Histone modification (blue)]; RNA Processing [GO: 0006396, RNA processing (red)]; and DNA Modifications [GO: 0006281, DNA repair (red) and GO: 0071103, Conformational changes (blue)].

To evaluate proteins that potentially engage with the core promoter of *CXXC5*, we used a promoter pull-down approach. Nuclear extracts of MCF7 cells were incubated overnight with a 5′-end biotinylated PCR amplicon containing Segment A (220 bp in length) or a fragment of Exon10 (220 bp) as control DNA followed by incubation with streptavidin-conjugated magnetic beads. Proteins bound to beads/DNA were then subjected to MS. Subtractive analysis of MS results obtained with proteins bound to beads, the control DNA, and Segment A revealed 94 proteins that specifically associate with the core *CXXC5* promoter (Fig. 4; Supplementary Information Fig. S4–S8; Supplementary Information Table 2). Analyses using STRING v11^62^ (https://string-db.org/) and DAVID^63^ (https://david.ncifcrf.gov/) databases suggest that Segment A associated-proteins are mainly grouped in the regulation of gene expression, which can further be sub-grouped into proteins as TFs and transcription co-regulatory proteins as well as proteins involved in histone/chromatin, DNA, and RNA processing (Fig. 4). Proteins identified as TFs include AFF1, ATF7, CCGBP1, CREB1, ELF1, MAZ, MGA, MYNN, NF1A, NF1B, PRDM10, TFAP2C, TPAP4, ZBTB2, ZBTB7A, ZBTB7B, ZNF596, and ZNF625. Transcription co-regulatory proteins comprise ANKRD12, ATXN7, BCOR, BRD2, BRD3, CBX8, MTA1, RBBP6, RB1, TADA2B, TRRAP, and WIZ. The group of proteins involved in the processing of RNA transcripts includes BUD31, CNOT1, DDX41, DDX49, DDX50, DDX54, RANBP2, and YBX1. The protein group associated with chromatin/histone binding, modifications, and organization as well as DNA conformational changes encompasses GATAD2A, INO80, JMJD1C, KAT2A, KDM1A, KDM2A, MCRS1, ORC5, RPA2, TAF6, TAF6L, and TOP3A.

To assess the binding of TFs obtained with the promoter pull-down approach as the putative binders to sequences of Segment A, we initially carried out bioinformatics analyses using the Cistrome (http://cistrome.org/) database, a resource of human and mouse cis-regulatory information derived from ChIP-seq, DNase-seq, and ATAC-seq chromatin profiling assays to map the genome-wide locations of transcription factor binding sites^64^. Due to the availability of information on TFs in Cistrome, the possible association of 16 TFs (AFF1, ATF7, CREB1, ELF1, MAZ, MGA, MYNN, NFIA, NFIB, PRDM10, RB1, TFAP2C, TFAP4, ZBTB2, ZBTB7A, and ZBTB7B) with Exon3 and surrounding sequences was analyzed with datasets generated by the use of MCF7 cells and/or of other cell lines for which datasets were available. Results revealed that while ATF7, CREB1, MGA, MYNN, NFIA, NFIB, ZBTB2, or ZBTB7B does not appear to interact with the Exon3 region, the association of ELF1, TFAP4, or TFAP2C with the region in cells seems to be dependent on tissue-of-origin. On the other hand, AFF1, MAZ, PRDM10, RB1, or ZBTB7A could be involved in the regulation of *CXXC5* expression in MCF7, and also in other, cells by interacting with the Exon3 region (Supplementary Information, Fig. S9).

### Interactions of TFs with Segment A *in cellula*

**Figure 5:**
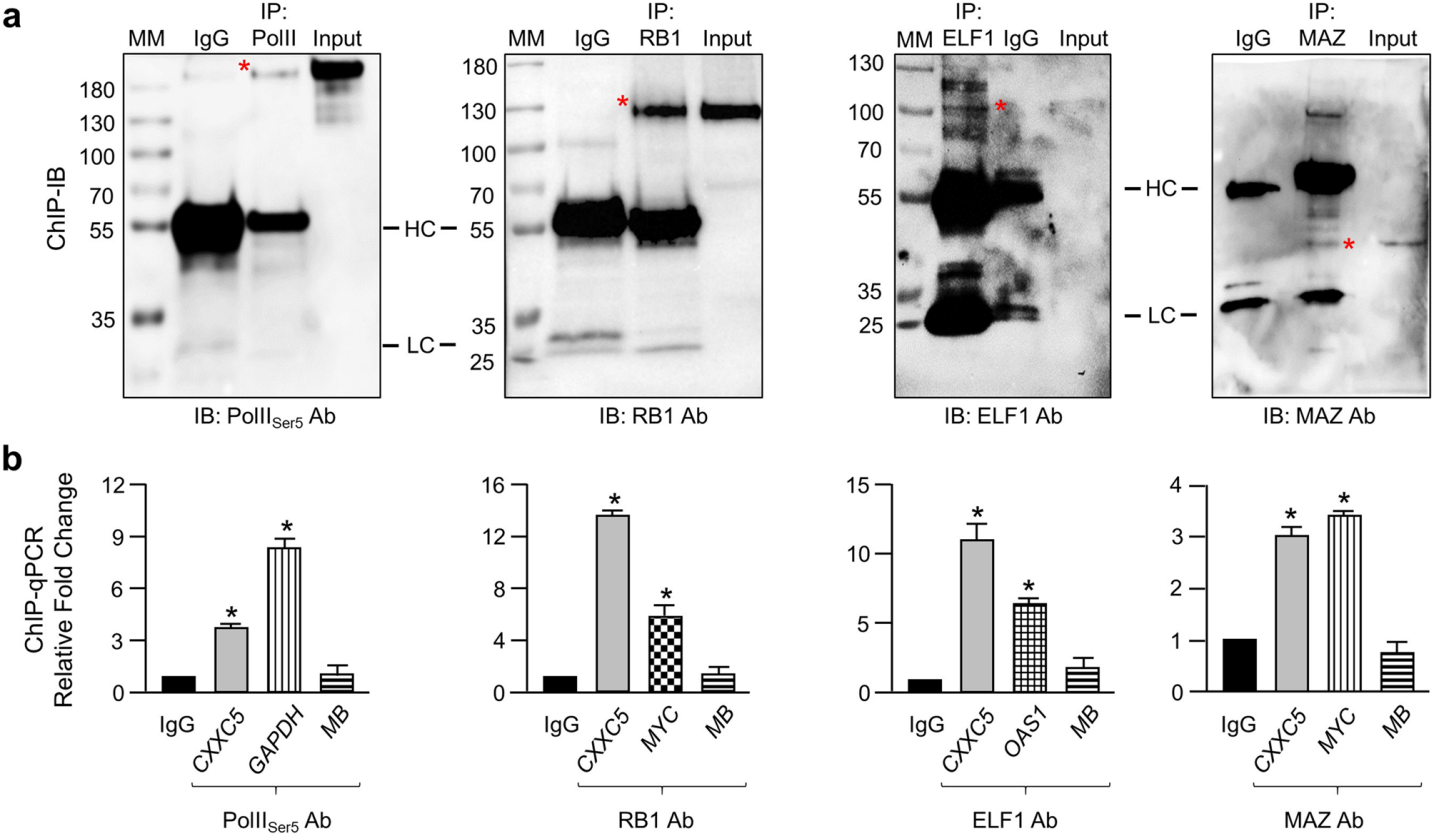
Assessing the interaction of Ser5P-PolII, RB1, ELF1, or MAZ with the core *CXXC5* promoter elements *in cellula*. (**a**) ChIP. Fragmented chromatin from MCF7 cells processed for ChIP was immunoprecipitated with a species-specific IgG or an antibody specific to Ser5P-PolII, RB1, or ELF1. Fragmented chromatin of MCF7 cells transiently transfected with MAZ_ΔN_ was also processed for ChIP to ensure that the MAZ antibody directed to the carboxyl-terminus of MAZ to be used in ChIP is capable of precipitating the endogenous MAZ. ChIP precipitates were subjected to SDS-PAGE for Ser5P-PolII and RB1 or to SDS-PAGE for ELF1 and MAZ_ΔN_ followed by IB using antibodies indicated for ChIP. Asterisk (*) indicates the protein of interest. Input, IgG together with heavy (HC) and light (LC) chains of IgG are indicated. Molecular masses (MM) in kDa are denoted. (**b**) ChIP-qPCR. While identical primer sets for each antibody were used in assessing the interaction of a transcription factor (TF) to Segment A as the core *CXXC5* promoter elements (CXXC5) or to the Exon2 of *Myoglobin* (MB) as a control, distinct primer sets were used for the promoter of a target gene of each TF. The mean ± SE of three independent experiments performed in triplicate is shown. Asterisk (*) indicates significant differences depicted as fold change compared to IgG.

MAZ binds to DNA sequences with high G nucleotide content^65,66^, which are abundantly present in the CXXC5 core promoter. ELF1, upon binding to DNA could regulate gene expressions through interaction with RB1^67^. RB1, which we identified here as one of the Segment A interacting proteins as well, indirectly associates with DNA through interactions with, for example, members of the E2F family proteins and hematopoietic transcription factors^68^. Based on these observations, we reasoned that ELF1 and MAZ could be involved in the regulation of the CXXC5 gene expression. We also carried out ChIP for RB1. To assess the possible presence of TFs on Segment A, we initially examined the efficiency of antibodies to precipitate the protein of interest with IB following ChIP (ChIP-IB) (Fig. 5a) and subsequently assessed the amount of isolated DNA with qPCR (ChIP-qPCR) (Fig. 5b) using primer sets specific for Segment A. We also used primers specific for the promoter of *OAS1* (2′-5′-Oligoadenylate Synthetase 1) with which ELF1 is shown to interact^67^ as control. Similarly, primer sets for the promoter of *MYC* (MYC Proto-Oncogene, BHLH Transcription Factor) were used to assess the interaction of, as shown previously, MAZ^69^ or RB1^70^ as control. We also used Exon2 of *MB* (Myoglobin) as control. In addition, ChIP using an antibody specific to CREB1 was conducted to ensure that CREB1 does not interact with the Exon3 region as the findings of the Cistrome database suggested.

**Figure 6:**
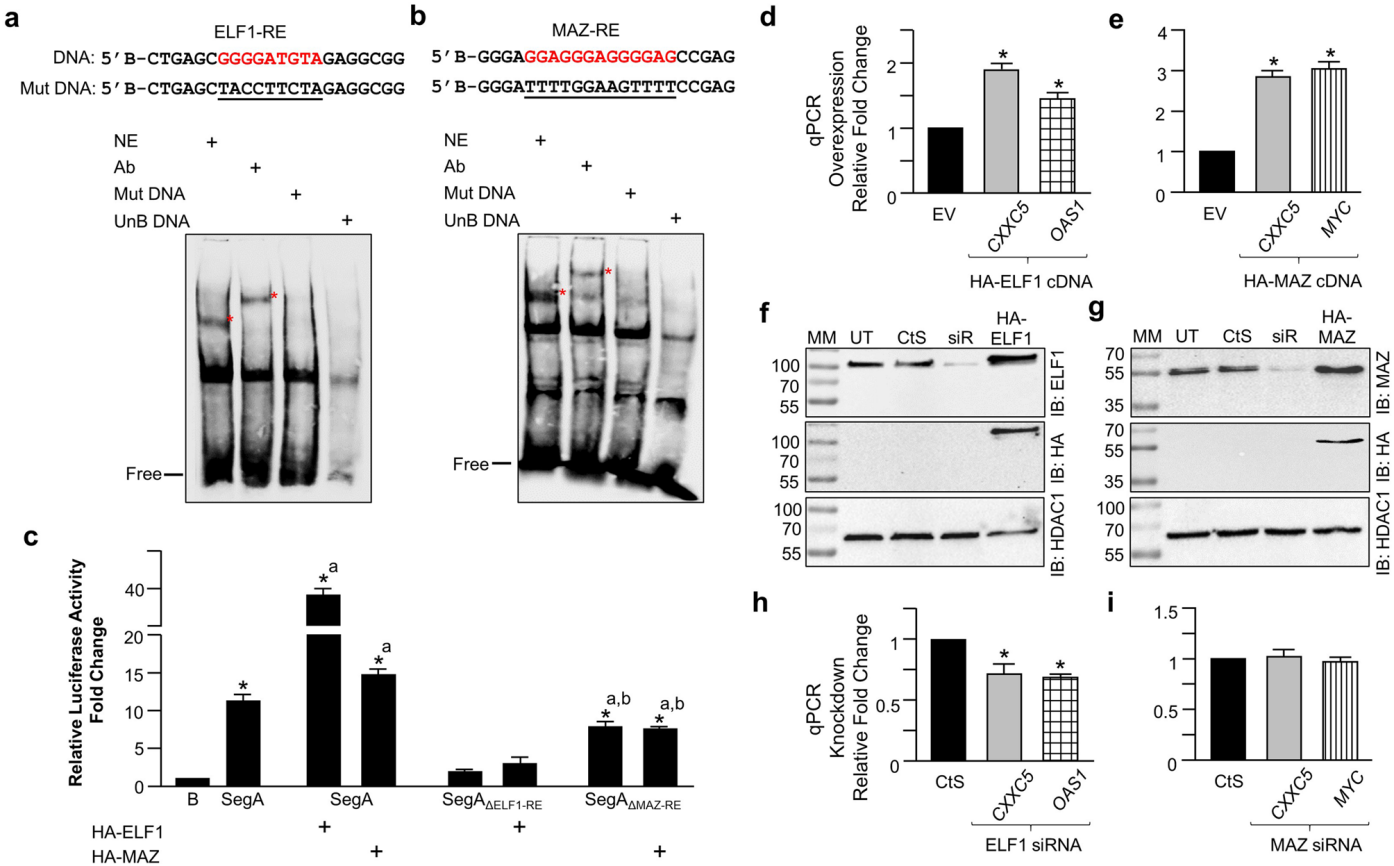
Assessing the interaction of ELF1 or MAZ with DNA. (**a**, **b**) Electrophoretic mobility Shift Assay (EMSA). 40 fmol of 5′ biotin-conjugated doubled stranded DNA fragment (shown is the upper strand) containing the wild type (red font) or mutant (underlined) ELF1 or MAZ response element (RE) derived from the *CXXC5* core promoter was incubated with nuclear extracts (NE, 45 µg) of MCF7 cells in the absence or presence (+) of an antibody (Ab) or 250-fold excess unbiotinylated DNA (UnB) with wild type RE. Free indicates the unbound biotinylated RE. Asterisks (red) indicate protein-bound DNA. (**c**) MCF7 cells were transfected with pGL3 bearing none (B), the wild-type (SegA), or Segment A with a deletion of ELF1-RE (SegA_ΔELF1-RE_), or MAZ-RE (SegA_ΔMAZ-RE_) as promoter driving *Luciferase* cDNA expression as the reporter without or with an expression vector bearing the HA-ELF1 or HA-MAZ cDNA for 24 h. The transfection efficiency was monitored by the co-expression of pCMV-Renilla Luciferase. Cellular extracts were then subjected to dual-luciferase assays. Asterisks (*) indicate significant differences depicted as fold change compared to B which was set to 1; whereas the superscript “a” denotes significant differences compared to SegA. (**d**, **e**) MCF7 cells were transfected with an expression vector bearing none (EV), HA-tagged ELF1, or HA-tagged MAZ cDNA. Isolated RNA was converted into cDNA libraries and followed by qPCR using either *CXXC5*-specific or *OAS1*-specific for ELF1 control or *MYC*-specific for MAZ control primers. Results were normalized with the *RPLP0* expression using the 2-^ΔΔCT^ method. (**f**, **g**) Nuclear extracts of untransfected (UT) or transiently transfected MCF7 cells with control siRNA (CtS), a pool of siRNA specific (siR) to ELF1 (**f**) or MAZ (**g**), or with an expression vector bearing the HA-ELF1 or the HA-MAZ cDNA for 48 h were subjected to IB using ELF1 or MAZ antibody. Membranes were then re-probed with the HA antibody. Molecular masses (MM) in kDa are indicated. (**h**, **i**) Total RNA from MCF7 cells transiently transfected cells with control siRNA (CtS), siRNA specific (siR) to ELF1 (**h**), or MAZ (**i**) were processed for and subjected to qPCR using primers specific to *CXXC5*, the *OAS1,* or *MYC*. Asterisks (*) denote significant differences depicted as fold change compared to CtS of three independent experiments.

Results revealed that ELF1 or RB1, as Ser5 phosphorylated PolII, indeed associates with Segment A, as each interacts with the promoter elements of the respective control gene but not with *MB* (Fig. 5b). MAZ synthesized endogenously in MCF7 cells displays electrophoretic mobility of about 57 kDa (Fig. 6g) that co-migrates with the heavy chain of IgG in immunoprecipitates (Supplementary Information, Fig. S10a). This renders the presence of MAZ in precipitates difficult to decipher. To ensure that the antibody, which recognizes sequences at the carboxyl-terminus of MAZ, precipitates the protein, we used an amino terminally truncated MAZ (MAZ_ΔN_) with an estimated MM of 37 kDa (Supplementary Information, Fig. S10b). MCF7 cells were transiently transfected with an expression vector bearing the HA-MAZ or HA-MAZ_ΔN_ cDNA. Cells were then subjected to ChIP-IB using the MAZ antibody. The presence of HA-MAZ_ΔN_ in the precipitates indicated that the antibody immunoprecipitates the MAZ protein (Supplementary Information, Fig. S10b). Based on this finding, we carried out ChIP of MCF7 cells using the MAZ antibody. qPCR results revealed that MAZ interacts with Segment A and the *MYC* promoter but not with Exon2 of *MB* (Fig. 5b).

CREB1 did not show an association with Segment A or *MB* but it interacted with the promoter elements of *CCNA2* (Supplementary Information, Fig. S10c,d), as shown previously^71^.

### Sequence motif analyses for ELF1 and MAZ on segment A

To examine binding sites for ELF1 or MAZ on Segment A, we performed sequence motif analyses using our motif analysis tool^72^ and the JASPAR (http://jaspar.genereg.net/) database^73^, which is a resource for curated, non-redundant TF-binding profiles stored as position frequency matrices (PFMs) for TFs. We identified potential binding sites for MAZ and ELF1 proteins in Segment A (Supplementary Information, Fig. S11a,b). Moreover, one of the characteristics of CGI promoters is the lack of sequence motifs for TATA-box or downstream promoter element (DPE) positioned at distinct locations relative to TSS that define non-CpG promoters^3,4,74^. Consistent with this, we found no such elements throughout the *CXXC5* locus including Segment A.

To corroborate the binding to the putative ELF1 or one of the MAZ motif of Segment A, we performed electrophoretic mobility shift (EMSA) assays, as we described previously^19^, using a 5′-end biotin-conjugated DNA substrate containing the ELF1 or MAZ binding motif present in Segment A and nuclear extracts of MCF7 cells (Fig. 6). When the DNA substrate for ELF1 (ELF1-RE, Fig. 6a) or MAZ (MAZ-RE, Fig. 6b) was incubated with nuclear extracts, a DNA–protein complex (asterisk) was observable on the gel. The electrophoretic migration of the protein–DNA complex with the inclusion of the ELF1 or MAZ antibody further retarded the migration. These results suggest that ELF1 or MAZ specifically interacts with the DNA substrate. The abrogation of the protein-DNA interaction with a DNA substrate bearing mutant sequences (Mut DNA) or with the inclusion of a 250-fold molar excess of the unbiotinylated (UnB DNA) ELF1-RE or MAZ-RE DNA further indicates that Segment A contains sequences for the binding of ELF1 or MAZ.

To assess the effects of ELF1 or MAZ on the expression of reporter enzyme driven by Segment A, we transiently transfected MCF7 cells with the expression vector bearing the HA-ELF1 or HA-MAZ cDNA for 24 h (Fig. 6c) Results indicated that ELF1 or MAZ enhances the enzyme activity compared to levels observed with the vector. Moreover, we observed reduced levels of reporter enzyme activity driven by Segment A with the deleted ELF1 (SegA_ΔELF1-RE_) or MAZ (SegA_ΔMAZ-RE_) motif compared to the native sequence in MCF7 cells whether or not cells transfected with the expression vector bearing the HA-ELF1 or HA-MAZ cDNA. These results indicate that Segment A contains sequences for the binding of ELF1 and MAZ critical for the promoter activity.

In assessing the effects of ELF1 or MAZ on *CXXC5* expression in a chromatin context, we transiently transfected MCF7 cells with the expression vector bearing none (EV), the HA-ELF1, or HA-MAZ cDNA for 48 h. HA-ELF1 (Fig. 6d) or HA-MAZ (Fig. 6e) augmented the expression of *CXXC5* as well as the corresponding control *OAS1* or *MYC* compared to the vector as assessed with RT-qPCR. Furthermore, the reduction of ELF1 protein levels (Fig. 6f) in transient transfections in MCF7 cells with a siRNA pool that targets ELF1 effectively attenuated the expression of *CXXC5* or *OAS1* (Fig. 6h). We also observed effective repression of the protein levels of MAZ by a MAZ-specific siRNA pool (Fig. 6g). Unexpectedly, however, the suppression of MAZ synthesis did not alter the *CXXC5* or the *MYC* expression (Fig. 6i). This suggests that MAZ at steady-state conditions in contrast to ELF1 may not contribute to *CXXC5* expression.

Thus, it appears that although ELF1 and MAZ participate in the expression of *CXXC5*, the contributory effect of these TFs on the *CXXC5* expression could be mechanistically distinct and context-dependent.

### Segment C may contain a G-quadruplex

Our reporter assays suggested that Segment C, which has a high G-C content, in the presence of other segments attenuates and alone represses the activity of the promoter driving the expression of the *Luciferase* cDNA as the reporter enzyme (Fig. 2).

G-rich sequences can self-associate into stacks of G-quartets to form complex structural motifs known as G-quadruplexes (G4s) which arise from Hoogsteen hydrogen bonding of four guanines arranged within a planar quartet (G‐quartet) linked by loop nucleotides^75,76^. Self‐stacking of G4 structure is further stabilized by monovalent cations, including K^+^^75,76^. G4s could play many essential functions including transcriptional events^75,76^. The consensus motif of G3 + N_1–7_G3 + N_1–7_G3 + N_1–7_G3 + N_1–7_ (G = guanine and N_1-7_ = 1–7 any nucleotide) is used to identify potential G-quadruplexes from the primary sequence^76^.

**Figure 7:**
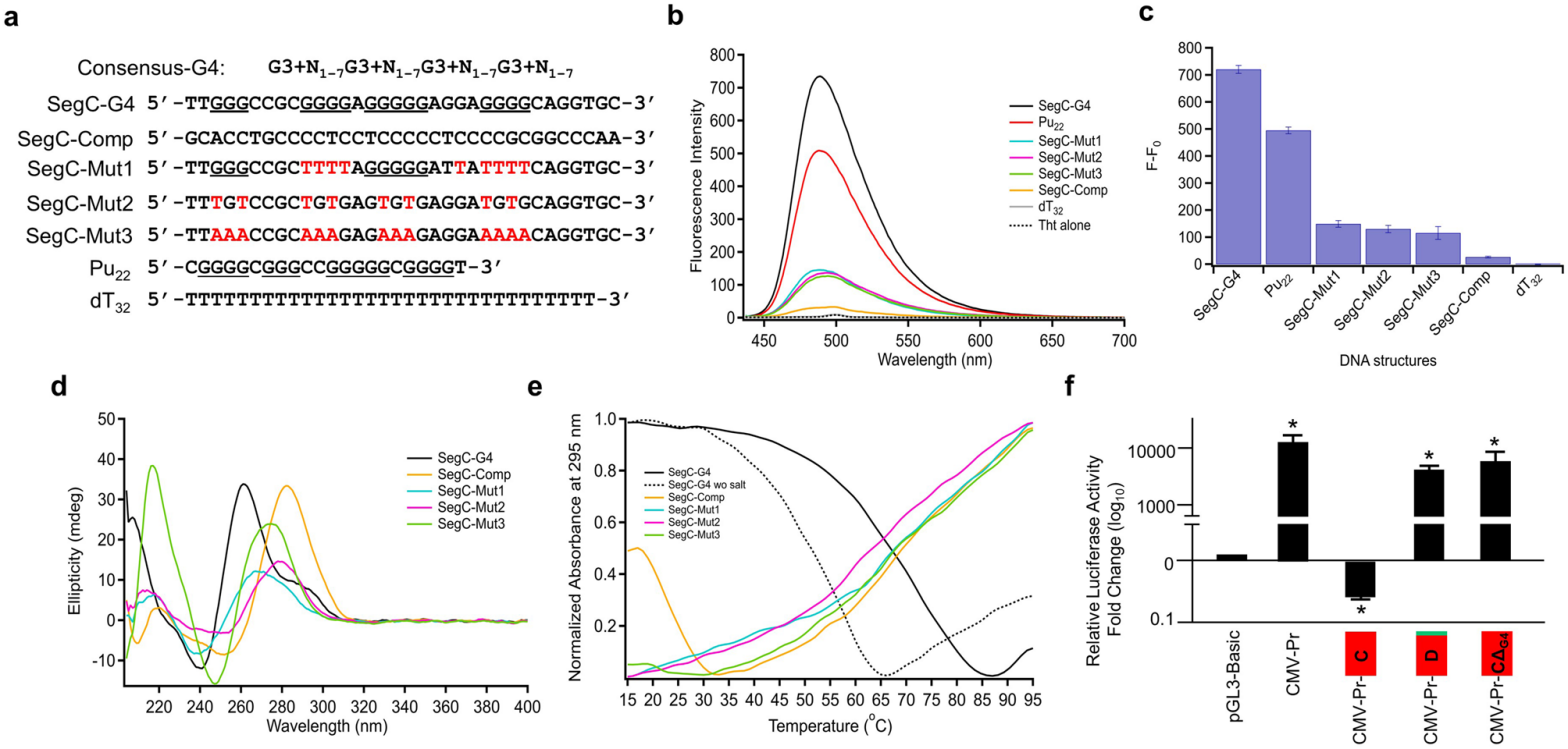
Segment C of Exon3 may contain a G-quadruplex. (**a**) Wild-type and mutant oligonucleotides (red font) of Segment C together with Pu_22_ of *VEGF* with possible G4 forming sequences (underlined) are shown. (**b**, **c**) Assessing the presence of a G4 structure in Segment C using Thioflavin T (ThT). (**b**) Fluorescence emission of ThT in the presence of selected nucleic acid sequences (**c**) bar graph of the change in ThT fluorescence in the presence of selected nucleic acid sequences. (**d**) CD spectra of Segment C oligonucleotides at 5 °C. (**e**) Thermal denaturation profile of Segment C oligonucleotides obtained from the change in UV–Vis absorbance at 295 nm between 15 to 95 °C. (**f**) MCF7 cells were transiently transfected with pGL3 bearing none (Basic-Luc), the CMV promoter without (CMV-Pr), or with the 3′-end genetically fused Segment C (CMV-Pr-C), Segment D (CMV-Pr-D), or Segment C bearing the G4 sequence deletion (CMV-Pr-CΔ_G4_). Reporter vectors drive the expression of the Firefly *Luciferase* cDNA expression as the reporter enzyme. The transfection efficiency was monitored by the co-expression of pCMV-RL that drives the expression of the *Renilla Luciferase* cDNA. 24 h after transfections, cellular extracts were subjected to dual-luciferase assays. Shown are the mean ± SE of three independent experiments performed in triplicate. Firefly/*Renilla Luciferase* activities are presented as fold change (log_10_) compared to pGL3-Basic, which is set to one. Asterisk (*) indicates significant differences from the Basic-Luc control.

Analysis of the sequence of Segment C strands with G4 prediction tools including G4Hunter^77^ (http://bioinformatics.ibp.cz/) and G4CatchAll^78^ (http://homes.ieu.edu.tr/odoluca/G4Catchall/) revealed the possible presence of a G4 on the positive strand (Fig. 7a). Based on these results, we initially wanted to explore the presence of a G4 structure in Segment C using Thioflavin T (ThT) which interacts selectively with G4s resulting in a significant fluorescent enhancement^79^. Incubation with ThT of the putative G4 sequence of Segment C (SegC-G4), 34 nt long, in the presence of 70 mM KCl led to a substantial fluorescence increase which was determined to be as F − F_0_ = 720 ± 15 (Fig. 7b,c). This increase in the fluorescence was comparable to that observed with the Pu_22_ sequence (F-F_0_ = 495 ± 13) present in the promoter region of the *VEGF* (vascular epithelial growth factor) gene, which was previously characterized to form a G4 structure^80,81^. These together with low fluorescence intensities of ThT at 488 nm with various mutant sequences of SegC-G4 designed to disrupt the G4 formation (SegC-Mut1-3), SegC complementary (SegC-Comp), or dT_32_ suggest the possible adaptation of a G4 structure by SegC-G4 sequence.

To verify that the SegC-G4 sequence indeed forms a G4 structure, we also used the Circular Dichroism (CD) approach, which is commonly utilized to determine the G4 topology of G-rich sequences^82,83^. The presence of a parallel G4 is characteristically associated with a positive band around 260 nm and a negative band around 240 nm. On the other hand, the formation of an antiparallel G4 reveals a positive band around 295 and 240 nm and a negative band near 260 nm. The hybrid type G4 structure is associated with a positive band at 290 nm together with a shoulder band at 260 nm and a negative band at around 240 nm^82–84^. In the CD spectrum of SegC-G4, a negative peak around 240 nm, a positive peak around 260 nm and another positive peak around 290 nm were observable (Fig. 7d). The presence of a negative peak around 240 nm and a positive peak around 260 nm is an indication of a parallel G4 structure. Besides, the presence of a positive peak around 290 nm suggests the existence of a second structure with a hybrid topology. The CD spectrum of SegC-Comp reveals an intense absorption maximum around 283 nm with a negative band around 254 nm, which might be correlated with the formation of an i-motif structure due to the C-rich content of the strand^85^. The mutant sequences (SegC-Mut1, SegC-Mut2, and SegC-Mut3) did not show the characteristic peak intensities of G4s. Compared to SegC-Mut1 and SegC-Mut2, the high intensity of the CD spectrum at 220 nm of SegC-Mut3 likely results from the A-rich content of this sequence^86^.

Thermal denaturation, using spectroscopic methods, offers an approach for measuring the stability of nucleic acid structures^87^. CD thermal denaturation experiments were conducted to further examine the G-quadruplex structure of SegC-G4. CD spectra were recorded as a function of temperature (between 15 °C and 95 °C) (Supplementary Information Fig. S12a). Thermal denaturation profile obtained by monitoring ellipticity change at 262 nm revealed a melting temperature (Tm) of 65 °C (Supplementary Information Fig. S12). Furthermore, thermal denaturation as a function of temperature recorded with changes in UV–Vis absorbance at 295 nm, a characteristic wavelength for G-quadruplexes, revealed also a characteristic thermal denaturation curve of a G4 structure^88^ in SegC-G4 (Fig. 7e). Additionally, a decrease in Tm of the SegC-G4 sequence from 65 to 45 °C in the absence of 70 mM KCl as the source of K^+^ for stabilization of G4 structures^75,76^ further indicates that the SegC-G4 sequence adopts a G4 conformation. On the other hand, in agreement with our CD data, no thermal denaturation curve was obtained for SegC-Mut1, SegC-Mut2, or SegC-Mut3 sequence (Fig. 7e).

Since the fusion of Segment C to the 3′-end sequence of the CMV promoter effectively repressed the reporter enzyme activity, in contrast to Segment D which has minimal effects, induced by the promoter (Fig. 2f), we wanted to assess whether the removal of this G4 sequence in Segment C would restore the CMV-driven enzyme activity. In MCF7 cells transiently transfected with the reporter plasmid bearing CMV promoter that drives the *Luciferase* enzyme cDNA as the reporter, the repression of the enzyme levels by the presence of Segment C (CMV-Pr-C) but not Segment D (CMV-Pr-D) was indeed effectively alleviated with the deletion of the G4 sequence in Segment C (CΔ_G4_) (Fig. 7f).

## Discussion

The majority of human gene promoters of housekeeping, developmental and tissue‐specific genes are located within unmethylated CGIs that display a chromatin state permissive for transcription which is initiated at multiple closely spaced TSSs by ‘broad or dispersed’ promoters in contrast to ‘focused or sharp’ non-CpG promoters of cell-type-specific genes within which a single TSS initiates transcription^3,4,56,74^. While sequence motifs for TATA-box and downstream promoter element (DPE) positioned at distinct locations relative to TSS tend to characterize non-CpG promoters, CGI promoters generally lack these elements^3,4,74^. We identified here transcript variant 2 with the highest expression level among transcript variants in MCF7 and HL60 cells as the main transcript of *CXXC5*. We also defined a DNA segment within and at the 5’ surrounding sequences of Exon3 as the core promoter region required for *CXXC5* expression. Based on DNA sequence composition and motifs, chromatin configuration of, and the presence of multiple TSSs together with an active PolII at the *CXXC5* promoter, we suggest that a CGI promoter drives the expression of *CXXC5*.

Transcription is the result of the integrated effects of multiple inputs mediated by TFs whose activities are dynamically modulated in response to internal and external signaling cascades. Studies indicate that due to a high CpG density^89^ and inherently unstable nucleosome architecture^90^, the chromatin accessibility of CGIs is critical for the binding of various transcription factors including the ZF-CXXC family proteins and the subsequent recruitment of DNA/histone modifiers and RNA polymerase machinery for transcription^3,4,74^. Our studies coupled with bioinformatics analyses suggest that AFF1, ELF1, MAZ, PRDM10, TFAP2C, TFAP4, and ZBTB7A transcription factors may be involved in the regulation of the *CXXC5* expression; of these, ELF1, MAZ, TFAP2C, TFAP4, and ZBTB7A appear to be capable of interacting with sequences enriched with C and/or G nucleotides within the nucleosome-free *CXXC5* promoter. We verified here that ELF1 and MAZ are critical components of the *CXXC5* expression by directly interacting with cognate sequence motifs present in the *CXXC5* promoter.

The regulation of *CXXC5* expression is likely multifactorial involving many transcription factors with activator or repressor functions responding to distinct signaling pathways. ELF1 (E74 Like ETS Transcription Factor 1), a ubiquitously expressed gene product, is a member of the ELF subfamily of the ETS transcription factor family which plays diverse roles in regulating many essential processes including embryonic development, cell cycle control, cell proliferation, apoptosis, cell migration, hematopoiesis, and angiogenesis^91,92^. ELF-1 interacts with a permutation of a consensus core sequence, AGGAA, (also, Supplementary Information, Fig. S11)^93^ on DNA and acts as an activator or repressor of target gene expressions. ELF1 could regulate gene expressions through interaction with RB1 as well^67^. The interaction of ELF1 with the pocket region of the hypo-phosphorylated RB1 was shown to be critical for gene expressions involved in cell cycle progression during T cell activation^94^. It is well established that hypo-phosphorylated RB1 restricts the ability of cells to replicate DNA by preventing G1 progression to the S phase of the cell cycle through repressing genes involved in cell cycle progression regulated by the E2F family and its obligatory dimerization partners DP family proteins through direct binding to E2F responsive elements^68,95^. Hyper-phosphorylation of RB1 leads to the dissociation of RB1 from E2F-DP complexes and subsequent activation of target gene expressions^68,95^. We observed here that ELF1 and RB1 are co-present as observed with promoter pull-down and each is enriched at the *CXXC5* promoter as assessed with ChIP. Moreover, our studies revealed that ELF1 interacts with an ELF1 sequence motif on Segment A and modulates the *CXXC5* expression. These observations, therefore, raise the possibility that the interaction between ELF1 and RB1 drives the *CXXC5* expression in a cell cycle-dependent manner. Indeed, our ongoing studies suggest that this might be the case.

As *ELF1*, *MAZ* is expressed ubiquitously in human tissues at varying levels^96^. MAZ is a six Cys2-His2 zinc finger transcription factor and recognizes a permutation of a cognate sequence of GGGAGGG (also, Supplementary Information, Fig. S11) primarily present on nucleosome-free regions in broad promoters in contrast to focused promoters^97^. MAZ is implicated in a wide range of transcriptional roles, including transcription initiation^69^, transcriptional pausing of PolII during transcription elongation^98^, alternative splicing^98,99^, and transcription termination leading to the activation of polyadenylation^69,100^. We observed here that MAZ is enriched at the *CXXC5* promoter assessed by ChIP, binds to MAZ binding motif by EMSA, and modulates the expression of *CXXC5* assessed by overexpression from the reporter promoter construct or the endogenous gene locus. These suggest that MAZ is a critical contributor for the expression of *CXXC5*. However, we also observed that the effective reduction of MAZ protein levels by a siRNA approach did not alter the *CXXC5* expression nor the expression of *MYC* used as the control, in contrast to the reduction in ELF1 protein levels which led to a decrease in the *CXXC5* expression. This suggests that MAZ may not be involved in the expression of *CXXC5* under steady-state conditions but is involved in the *CXXC5* expression in response to a signaling pathway. It is also likely that the decrease/absence of MAZ might be compensated with other transcription factors that bind similarly to DNA binding motifs of MAZ. Indeed, the sequences of the binding sites for MAZ and SP1 (Specificity factor 1), which are often found within the same gene, are very similar: GGGAGGG and GGGCGG, respectively^65,66^. Studies further showed that SP1 binds, and competes with MAZ for binding, to the same GC-rich DNA-binding sites^101^.

Although how ELF1 or MAZ modulates *CXXC5* expression is unclear, alterations in histone modifications, upon binding to DNA, of target gene promoters appear to be critical for gene expressions^65,102^. MAZ, for example, represses transcription by recruiting histone deacetylases including HDAC1, HDAC2, and HDAC3^65^. Moreover, the interaction of FAC1 (Fetal Alzheimer's clone 1), a truncated isoform of the chromatin remodeler BPTF (bromodomain and PHD domain transcription factor), with MAZ is shown to alter the transcriptional activity of the protein^103^. It is therefore likely that upon association with DNA, MAZ and/or ELF1 directly or through co-regulatory proteins interact with histone modifiers, as we find here with the pull-down assay of the association of histone acetyltransferase KAT2A as well as histone demethylases JMJD1C, KDM1A, and KDM2A with Segment A, and establishes a chromatin state and structure permissive/restrictive for the transcriptional regulation of *CXXC5*.

While Segment A of the first exon of transcript variant 2 constitutes the core promoter for the *CXXC5* gene, the surrounding regions may contribute to gene expression as they contain potential binding motifs for various transcription factors (data not shown) as well as structural features, exemplified here with the presence of a G4 conformation in Segment C. As non-canonical nucleic acid secondary structures formed within G-rich sequences in both DNA and RNA, G4s are widely found in promoter regions, immunoglobulin class switch regions, ribosomal DNA, mitochondrial DNA, replication initiation regions as well as in the extended repeat sequences in various pathologies^104,105^. G4s play fundamental roles in transcription, replication, genome stability, and epigenetic regulation as well as post-transcriptional events including RNA transport, localization, and translation^104,105^. G4 structures could act as modifiers of various TFs, as exemplified with p53^106^, at target promoter sites in the regulation of gene expression. The abilities of various proteins including helicases, chromatin/histone modifiers, and transcription factors to interact with G4 may be critical for the dynamic regulation of gene expressions. For example, the binding of nucleolin to the nuclease hypersensitive element III_1_ (NHE III_1_) of *MYC* induces the formation of a G4 structure and reduces the *MYC* transcription^107^; the binding of NME (non-metastatic cell 2; Nucleoside diphosphate kinase B), on the other hand, unfolds the G4 structure and promotes the transcription of *MYC*^108^. MAZ is also shown to bind to secondary DNA structures including G4s, which appears to be critical for transcriptional events of target genes^109–111^. In addition to Segment A, we also observe adjacent binding motifs for MAZ (GGGGAGGGGGAGGAGGG) in Segment C (Fig. 7A; Supplementary Information, Fig. S11). This raises the possibility that the interaction of MAZ with Segment C could also modulate the transcription of *CXXC5* by forming or resolving the G4 structure.

Although promoters constitute the key platform for the assembly of pre-initiation complexes to mediate the directionality and accuracy of transcription initiation, enhancers are DNA regulatory elements that determine spatio-temporal expression even over long distances regardless of its orientation to the core promoter^112,113^. It is well established that enhancers acting as binding targets for lineage-specific TFs are critical components of transcription by establishing proximity interactions with promoters in a cell-type-specific manner^112,113^. Given the fact that transcription requires dynamic protein–protein interactions and subsequent multistep ordered assembly of protein complexes within a temporally modulated chromatin architecture, a better understanding and delineation of mechanistic features of the *CXXC5* expression in response to distinct signaling pathways including retinoic acid^11^, TGF-β^12^, BMP4^13,14^, Wnt3a^15–17^ and estrogen^18–20^ would be a valuable input for both physiology and pathophysiology.

## Materials and methods

### Biochemicals

Restriction and DNA modifying enzymes were obtained from New England Bio-Labs (Beverly, MA, USA) or ThermoFisher (ThermoFisher, Waltham, MA, USA). Chemicals were obtained from Sigma-Aldrich (Germany) or ThermoFisher. Pageruler Prestained Protein Ladder (ThermoFisher; 26616) or Pageruler Plus Prestained Protein Ladder (ThermoFisher; 26620) was used as the molecular mass (MM) marker.

### Cell culture and transfection

MCF7 cells were grown in phenol red-free, high glucose (4.5 g/L) containing Dulbecco’s Modified Eagle’s Medium (DMEM, Lonza, Belgium, BE12-917F) supplemented with 10% fetal bovine serum (FBS, Lonza), 1% l-Glutamine (Lonza, BE17-605E) and 1% Penicillin/Streptomycin (Lonza, Belgium) as described previously^19,29,114^. HL60 cells derived from acute promyelocytic leukemia were grown in phenol red-free, low glucose (1 g/L) containing DMEM supplemented with 10% fetal bovine serum, 1% L-Glutamine (Lonza, BE17-605E), and 1% Penicillin/Streptomycin. MCF7 cells were transiently transfected with Turbofect transfection reagent (R0533; ThermoFisher) for 48 h if not otherwise specified. Protein concentrations in extracts were assessed with a Bradford protein assay kit (Bio-Rad Life Sciences; 5000001).

### Engineering of reporter vectors

To assess the promoter activity of the genomic region of *CXXC5*, we generated *Luciferase* reporter vectors bearing a DNA fragment containing the putative promoter elements of the CXXC5 gene. We used the pGL3-Basic Luciferase Reporter vector that bears the *Firefly Luciferase* cDNA as the reporter enzyme (Promega Corp., Madison, WI, USA). For the engineering of the reporter vector bearing the putative *CXXC5* promoter containing genomic region, a DNA fragment of 1975 bp of the *CXXC5* locus (GRCh38.p12 Primary Assembly, chromosome 5: 139647220–139649173) generated by PCR using the genomic DNA of MCF7 cells as a template was inserted into the pGL3-Basic vector with appropriate restriction enzymes. To increase the resolution of the putative *CXXC5* promoter region, we carried out deletions from both the 5’ and 3’ ends of the region by PCR and inserted them into the pGL3-Basic vector with appropriate restriction enzymes. All constructs were sequenced for PCR fidelity. TFF1 (also known as the pS2 gene) is a well-studied estrogen-responsive gene^19,52^. The human TFF1 gene confers E2 responsiveness through the binding of ER to a non-consensus ERE^115–117^. The pGL3-TFF1 reporter construct is responsive to E2 in transiently transfected cells synthesizing estrogen receptor(s) exemplified with MCF7 cells^19,118,119^. We used the pGL3-TFF1 reporter vector bearing the estrogen-responsive TFF1 gene promoter as control under the steady-state cellular growth condition in that the growth medium contains unprocessed fetal bovine serum (FBS) as opposed to charcoal–dextran treated FBS to remove endogenous steroid hormones, including estrogens. In transfections, transfection efficiency was monitored with a reporter vector bearing CMV promoter that drives the expression of the *Renilla Luciferase* cDNA (pCMV-RL, Promega, Corp., Madison, WI, USA). For luciferase studies, cells, 4 × 10^4^ cells/well, were seeded in 48-well plates for 48 h. Cells were then transiently transfected with a 125 ng reporter vector together with 0.5 ng pCMV-RL using Turbofect. Luciferase assays were performed with a Dual-Luciferase Assay Kit (Promega, Corp., Madison, WI, USA) as described previously^19^.

### PCR and RT-qPCR

Isolated total RNA from MCF7 or HL60 cells was used for the cDNA synthesis (The RevertAid First Strand cDNA Synthesis Kit, Thermo-Fisher) and transcript variant identification was carried out by PCRs with transcript variant-specific primer sets (Supplementary Information, Table 1) followed by TA-cloning into the pGEM-T vector (Promega, Corp., Madison, WI, USA) and sequencing (PRZ Biotechnology, Ankara, Turkey). Transcript variant quantification studies were carried out with RT-qPCR. The SsoAdvanced Universal SYBR Green Supermix (Bio-Rad Life Sciences Inc., Hercules, CA, USA), transcript variant-specific qPCR primers (Supplementary Information, Table 1), and DMSO when it is necessary, were used. Expression levels of transcript variants were assessed with the efficiency corrected form of the 2^−ΔCT^ method^120^ and normalized using the *RPLP0* expression levels. Relative expression levels of *CXXC5*, and ELF1- or MAZ-regulated genes were assessed using 2^−ΔΔCT^ method^120^ and normalized using the *RPLP0* expression levels. Results were adjusted to the expression level of transcript variant 1, which was arbitrarily chosen, as one. In all RT-qPCR experiments, MIQE Guidelines were followed^121^.

### 5′ or 3′ rapid amplification of cDNA ends (5′RACE and 3′RACE)

For 5′RACE or 3′RACE studies, we used the RiboMinus Human/Mouse Transcriptome Isolation Kit (#K155001, Thermo Scientific, USA) to enrich the mRNA concentration in the total RNA population from MCF7 cells. We performed rRNA removal according to the manufacturer’s instructions. The method is based on the selective depletion of the rRNAs by the hybridization of rRNA to Locked Nucleic Acid (LNA) probes conjugated to magnetic beads. LNA referred to as inaccessible RNA is a modified RNA nucleotide that significantly increases the hybridization properties to DNA or RNA. After the hybridization, LNA probe bound rRNAs were captured with the help of a magnetic stand and the supernatant containing largely mRNAs depleted of rRNA was recovered. Phenol:chloroform:isoamyl alcohol and ethanol precipitation was then used for the purification of mRNAs.

For the identification of 5′- and 3′-ends of *CXXC5* transcripts as well as of *TFF1* as control, we used the FirstChoice RLM-RACE Kit (AM1700, ThemoFisher Scientific, USA) as directed by the manufacturer. For 5’RACE, in brief, purified mRNA (500 ng) was subjected to calf intestinal alkaline phosphatase (CIP) at 37 °C for one hour. Following the termination of the CIP reaction, RNA was extracted with phenol:chloroform:isoamyl alcohol, and ethanol precipitated. Resuspended RNA in nuclease-free water was then treated with tobacco acid pyrophosphatase at 37 °C for one hour and subjected to the ligation using a 5'RACE adapter and T4 RNA ligase. Ligated RNA products were subsequently reverse transcribed with M-MLV reverse transcriptase (M-MLV-RT) using random decamers at 42 °C for one hour. An aliquot of reactions was then used for outer 5′ RLM-RACE PCR with a 5′RACE CXXC5- or TFF1-specific 5′RACE outer primer (Supplementary Information, Table 1). Outer PCR reaction was followed by Inner 5′ RLM-RACE PCR using 5'RACE CXXC5- or TFF1-specific inner primer and a 5'RACE inner primer (Supplementary Information, Table 1). For 3′RACE, purified mRNA was subjected to reverse transcription using M-MLVRT and 3′RACE Adapter provided by the kit at 42 °C for one hour. An aliquot of the reaction was used for PCR using 3′ RACE CXXC5- or TFF1-specific outer primer and 3′RACE RLM adapter outer primer (Supplementary Information, Table 1). For both 5′RACE and 3′RACE, PCR amplicons were PCR column purified (DNA Clean & Concentrator-25, D4033, Zymo Research, Irvine, CA, USA), cloned into a vector, and sequenced (PRZ, Turkey).

### Northern blotting

#### Preparation of biotin-tagged probes

PCR amplicons for targeted identification of Exon3, Exon boundaries of Exon10 and Exon11 of *CXXC5* as well as of the *GAPDH* cDNA fragment containing Exon5-8 were cloned into a vector. We then used biotin-conjugated vector-specific primers (Supplementary Information, Table 1) for the PCR amplification of double-stranded probe sequences. To examine the presence of *CXXC5* transcripts in MCF7 and HL60, a northern blot assay was performed by the NorthernMax Kit (Thermo-Fisher, AM1940) according to the manufacturer’s instructions. In brief, 10 µg RiboMinus-treated RNA samples as well as 3 µl of RNA ladder (RiboRuler High Range RNA Ladder, # SM1821, Thermo Scientific, USA) were mixed with 3 volumes of formaldehyde loading dye and incubated at 65 °C for 15 min. Samples were then loaded to a denaturing agarose-LE gel and electrophoresed for 2 h and were transferred onto a positively charged PVDF membrane (Sigma-Aldrich, Roche, #11209272001). Transferred mRNAs were then cross-linked to the membrane with a UV transilluminator (312 nm wavelength) for 10 min. Membranes were placed into 15 ml sterile falcon tubes and pre-hybridization was initiated using 6 ml of ULTRAhyb buffer pre-heated to 42 °C in a vertical rotator for 40 min. Biotin-tagged probes were diluted tenfold with 10 mM EDTA containing TE buffer to a final volume of 100 µl and denatured at 90 °C for 10 min. The mixture was immediately added onto the membranes in falcon tubes and placed in the oven for hybridization overnight. Membranes were then washed twice with a low stringency buffer at RT for 5 min, followed by washing twice with a high stringency buffer for 15 min at 42 °C. Membranes were subsequently subjected to the Chemiluminescence Nucleic Acid Detection Module (89,880, Thermo Scientific, USA) which enables the detection of biotin-tagged nucleic acids with the utilization of HRP-conjugated streptavidin, according to the manufacturer’s instructions. Membranes were visualized with the Chemidoc MP system (Bio-Rad, USA).

#### Bisulfite conversion of DNA for methylation analysis

For methylation analyses of CpG rich regions of the *CXXC5* locus, we subjected 500 ng of isolated gDNA from MCF7 cells to bisulfite reaction for the conversion of unmethylated cytosine residues to uracil using the EZ-DNA Methylation Lightning Kit (Zymo Research, #D5030). The bisulfite converted DNA was subsequently used as the template for PCR with bisulfite primers (Supplementary Information, Table 1) designed by the use of the MethylViewer^122^ tool (http://www.insilicase.com/Desktop/Methyl-Viewer.aspx). It should be noted that bisulfite primers with no or at most one CpG position conserved were readily designable for sequences of − 930 to + 103th nucleotide of Exon3 of the *CXXC5* gene. However, due to the very high number of CpG positions at the center of Exon3 until the end of Exon4, designing bisulfite primers were precluded. We instead designed methyl-specific primers, which were based on methylated and unmethylated DNA sequences generated after bisulfite conversion. Methyl-specific PCRs were carried out with primer sets containing three or more CpG sites and these regions were PCR amplified as 3 overlapping segments. PCR was carried out with 2.5 units LongAmp Taq Polymerase (NEB, M0323) in 50 µl total reaction containing 0.5 µM forward and reverse primers. Template DNA was added to the reaction at 90 °C to prevent nonspecific primer annealing and the first two cycles of the reaction were carried out only in the presence of reverse primer complementary to the sense strand to avoid the formation of primer dimers. The subsequent 10 cycles were performed 5 °C above the annealing temperature followed by 30 cycles of PCR^123^. In all PCRs with bisulfite-converted DNA templates, we also used a bisulfite-converted “Universal Methylated Human DNA Standard” (Zymo Research, D5011) as control. PCR amplicons with expected sizes were excised from agarose gels, purified, and cloned into the pGEM-T vector (Promega Corp., Madison, WI, USA) by TA-cloning and sequenced (PRZ Biotechnology, Ankara, Turkey). Sequences were analyzed using the QUMA^57^ tool.

#### Assessing nucleosome occupancy with micrococcal nuclease assay

To assess nucleosome occupancy at the putative promoter region of the *CXXC5* gene, MCF7 cells, grown in six-well plates for 48 h were fixed with 2% formaldehyde in 1xPBS for 15 min at RT with gentle shaking. To quench the formaldehyde, 0.125 M Glycine (BioShop, #GLN002) solution was added and cells were incubated for 10 min. Cells were then washed with 1xPBS twice and 0.4% Triton X-100 was added onto the cells for permeabilization. Cells were subsequently washed twice with 1 × Micrococcal Nuclease (MNase) buffer (50 mM Tris–HCl, 5 mM CaCl_2_, pH 7.9 at 25 °C). 500 or 1000 Gel Units (GU) of MNase (NEB, #M0247S) diluted in MNase buffer was added and cells were incubated for 30 min at 37 °C for chromatin digestion. Digestion reaction was stopped by the addition of 10 mM EGTA (Merck, 67,425). Cells were then collected in a lysis buffer containing 1% SDS, 10 mM EDTA, 50 mM Tris–HCl pH 8.0, 0.5 mM PMSF, Protease Inhibitor (PI) by scraping. To reverse crosslink, 200 mM NaCl as the final concentration was added onto cell lysates and incubated at 95 °C for 5 min. To digest RNA, 0.2 mg/ml RNAse A (Thermo Scientific, EN0531) was added onto the lysate and incubated at 37 °C for 30 min. Lastly, to digest cellular proteins, 0.25 mg/ml Proteinase K (Thermo Scientific, AM2542) was added into the lysate and incubated overnight at 65 °C. DNA was subsequently purified by phenol:chloroform:isoamyl alcohol (VWR, #K-169) and ethanol precipitation. To analyze the digestion pattern, purified DNA was loaded on an agarose gel and bands corresponding to the tri-nucleosomal and mono-nucleosomal DNAs were excised and gel purified using Zymoclean Gel DNA Recovery Kit (D4001). 50 ng of purified DNA or uncut genomic DNA was used as a template in PCR reactions to assess nucleosome occupancy. For the initial scanning, five overlapping regions (depicted as T1-5, Fig. 3) were amplified from the tri-nucleosomal DNA template. For further verification, 3 sub-regions (depicted as M1-3, Fig. 3) were analyzed from the mono-nucleosomal DNA by PCR.

#### Chromatin immunoprecipitation assay

ChIP assays were carried out as described previously^19,119,124^. In brief, MCF7 cells were grown in medium supplemented with 10% FBS in T75 tissue plates for 48 h were fixed with 1% formaldehyde at RT for 15 min and lysed with Nuclei Lysis Buffer containing 1% SDS, 10 mM EDTA, 50 mM Tris–HCl pH 8.0, 0.5 mM PMSF, 1X PIC (Roche) and actively sonicated for 20 min. Cell debris was pelleted and the supernatant was collected. After pre-clearing of nuclear extracts, the supernatant was incubated with a species-specific (Mouse or Rabbit) IgG (Santa Cruz Biotechnolohy Inc., Santa Cruz, CA, USA), PolII antibody (POLR2A, CTD4H8; Santa Cruz) for the precipitation of hypo- and hyper-phosphorylated PolII; or Ser5-PolII (POLR2A, Phospho-Rpb1 CTD, D9N5I, Cell Signaling Technology, Beverly, MA, USA) for the precipitation of Ser5 phosphorylated PolII overnight. Nuclear extracts were also incubated with an antibody specific to CREB1 (D-12, Santa Cruz sc-377154), ELF1 (B-9, Santa Cruz, sc-133210), MAZ (133.7, Santa Cruz, sc-130915), or RB1 (Retinoblastoma, 4H1, Cell Signaling Technology, Beverly, MA, USA, #9309) overnight.

ChIP assays for total Histone 3 and H3K4me3 were performed following MNase digestion (as described in Micrococcal Nuclease Assay section) and sonication for 5 min followed by the incubation with a species-specific IgG, histone H3-1B1B2 mouse mAb (Cell Signaling Technology, #14269), or Histone 3 trimethylation at lysine 4, Tri-Methyl-Histone H3 (Lys4)-C42D8 Rabbit mAb (Cell Signaling Technology, #9751).

Samples were then subjected to immunoprecipitation with Protein G-coupled magnetic beads (New England BioLabs) for anti-mouse antibodies or Protein A/G coupled magnetic beads (New England BioLabs) for anti-rabbit antibodies. After washes, de-crosslinking, and protein digestion, DNA was recovered with phenol:chloroform: isoamyl alcohol followed by ethanol precipitation. Samples (1 µl of 60 µl elution) were subjected to qPCR using ChIP primers (Supplementary Information Table 1) specific to the putative *CXXC5* promoter, the promoter of *OAS1* (2′-5′-Oligoadenylate Synthetase 1), *MYC* (MYC Proto-Oncogene, BHLH Transcription Factor), or *GAPDH* (as the positive control, for PolII occupancy), or the Exon2 of *MB* (*Myoglobin*) as a negative control.

### Pull-down assay

#### Nuclear protein extraction

MCF7 cells grown in T75 flasks were trypsinized and collected by centrifugation at 1000×*g* for 5 min at 4 °C. Pellet was washed with ice-cold PBS twice and packed cell volume (PCV) was determined. Cells were resuspended in 1xPCV of Buffer A [Swelling Buffer: 10 mM HEPES pH 7.9, 1.5 mM MgCl_2_, 10 mM KCl, 0.5% NP-40, freshly added; 0.5 mM Phenylmethylsulfonyl Floride (PMSF), 0.5 mM DTT, and 1xProtease Inhibitor (PI)] and rested on ice to allow cells to swell. Cells were then lysed by passaging 25 times through a 25-gauge needle. Lysed cells were centrifuged to pellet crude nuclei at 12,000×*g* for 20 s at 4 °C. The crude nuclear pellet was washed twice with 1xPCV of IB [ice-cold Wash Buffer: 10 mM HEPES pH 7.9, 1.5 mM MgCl_2_, 10 mM KCl, freshly added; 0.5 mM Phenylmethylsulfonyl Floride (PMSF), 0.5 mM DTT, and 1xProtease Inhibitor (PI)]. After centrifugation, the pellet was resuspended in 2/3 PCV of Buffer B [ice-cold, 20 mM HEPES pH 7.9, 1.5 mM MgCl_2_, 420 mM KCl, 0.2 mM EDTA, 2.5% glycerol, freshly added; 0.5 mM Phenylmethylsulfonyl Floride (PMSF), 0.5 mM DTT, and 1xProtease Inhibitor (PI)] and rested on ice for 30 min with occasional agitation. Samples were then centrifuged for 5 min at 4 °C. Supernatant was then diluted isovolumetrically to decrease the salt concentration to 125 mM with Buffer D [ice-cold, 20 mM HEPES pH 7.9, 1.5 mM MgCl_2_, 100 mM KCl, 0.2 mM EDTA, 10% glycerol, freshly added; 0.5 mM Phenylmethylsulfonyl Floride (PMSF), 0.5 mM DTT, and 1xProtease Inhibitor (1xPI)].

#### Promoter pull-down

Based on luciferase reporter results, we amplified a 220 bp in length DNA fragment by PCR from the genomic DNA of MCF7 cells that includes the *CXXC5* promoter (Segment A; − 117 to + 103, + 1 being the first nucleotide of the annotated Exon3) and inserted it into a vector with appropriate restriction enzyme cut sites. Similarly, a 220 bp in length DNA fragment within Exon10 of the *CXXC5* gene as control DNA was cloned into the vector. 5′ end-biotinylated forward and reverse primers specific to the vector were then used for the amplification of Segment A and the control Exon10 DNA sequences by PCR. Biotinylated double-stranded PCR amplicons were recovered from agarose gels with Zymoclean Gel DNA Recovery Kit (Zymo Research).

Streptavidin magnetic beads (SMB, NEB) were blocked using 2% BSA in PBS for 2 h at 4 °C followed by washes with 1xPBS twice. The blocked SMBs were resuspended in 200 µl PBS containing 0.5 mM Phenylmethylsulfonyl Floride (PMSF), 0.5 mM DTT, and 1xPI, which were then mixed with one ml of nuclear extracts for pre-clearing for 1 h at 4 °C in 300 µl 1xPBS. Subsequently, the pre-cleared nuclear extract was divided into three equal (about 400 µl) aliquots. One aliquot of the extract was then mixed with 10 µg biotinylated double-stranded Segment A, control DNA or beads alone in the presence of 10 µg of Poly[d(I-C)] to form the protein-DNA complexes overnight at 4 °C on a rotator. The SMB–DNA–protein mixtures were subsequently washed with 1xPBS three times for 5 min each and resuspended in 200 µl 1xPBS for Mass Spectrometry (MS) analyses.

#### Protein identification by mass spectrometry

MS analyses of two biological replicates were carried out at the Koç University Proteomic Facility (Istanbul, Turkey). The SMB–DNA–protein mixtures were washed with 50 mM NH_4_HCO_3_, followed by reduction with 100 mM DTT in 50 mM NH_4_HCO_3_ at 56 °C for 45 min, and alkylation with 100 mM iodoacetamide at RT in the dark for 30 min. MS Grade Trypsin Protease (Pierce) was added onto the beads for overnight digestion at 37 °C (enzyme to protein ratio of 1:100). The resulting peptides were purified using C18 StageTips (ThermoFisher). Peptides were analyzed by online C18 nanoflow reversed-phase HPLC (2D nanoLC; Eksigent) linked to a Q-Exactive Orbitrap mass spectrometer (ThermoFisher). The data sets were searched against the human SWISS-PROT database version 2014_08. Proteome Discoverer (version 1.4; ThermoFisher) was used to identify proteins. The final protein lists were analyzed using the STRING v11^125^ and DAVID^63,126^ databases.

### In silico analysis of TF motifs for the *CXXC5* locus

#### Binding motifs for transcription factors

To find TF binding motifs, we developed a motif search tool^72^ using all the available ChIP-Seq datasets at the Cistrome^64^ database. This tool obtains: (1) a set of binding locations on a sample of Chip-Seq reads using MACS2 peak locations, (2) the reference sequence of the genomic locus to analyze, and (3) the binding motifs for a specific Transcription Factor from the JASPAR^73^ database as inputs. The program conducts and approximates string search on binding locations of the reference sequence using the consensus binding motif as the query sequence. The program generates both the forward and reverse strand hits which are ranked to a logarithmic sequence similarity score on binding locations.

#### Electrophoretic mobility shift assay (EMSA)

EMSA was conducted as described previously^19^. 5′ end biotin-labeled oligomers bearing ELF1 or MAZ binding motif sequence were purchased from Integrated DNA Technologies (IDT Europe; Belgium) and annealed. Double-stranded DNA fragments were incubated in the presence or absence of (45 μg) nuclear extracts for 15 min. Reactions were further incubated without or with the ELF1- or MAZ-specific antibody for another 15 min. Samples were subjected to electrophoresis on 5% non-denaturing polyacrylamide gel. Gel contents were subsequently electrophoretically transferred to a nylon membrane and processed for EMSA using the LightShift Chemiluminescent EMSA kit (Thermo-Fisher). In brief, the membrane was UV cross-linked and blocked for non-specific binding using a blocking buffer. The membrane was then probed with Streptavidin–Horseradish Peroxidase Conjugate in the blocking buffer for image development. Images were then captured using ChemiDoc Imaging System (Bio-Rad).

#### Immunoblotting (IB)

IB was carried out as described previously^19,29^. Briefly, cells were grown in six-well tissue culture plates in medium supplemented with 10% FBS for 48 h and transfected with a siRNA pool targeting ELF1 or MAZ and an expression vector bearing the HA-tagged ELF1 or the MAZ cDNA for 48 h. Cells were collected and protein isolation was performed using the NE-PER protein extraction kit (Thermo-Fisher). Protein concentration was determined using Bradford Proteins Assay (Bio-Rad). Nuclear extracts were subjected to denaturing SDS-PAGE, transferred to a membrane and proteins were probed with an antibody specific to ELF1 or MAZ followed by a secondary antibody conjugated with the horseradish peroxidase (Advansta). The membranes were then re-probed with the HA antibody (Abcam, ab9110) and subsequently with an HDAC1 antibody (Abcam, ab19845) as a loading control. Images were developed using the ECL-Substrate (Advansta) and captured with ChemiDoc Imaging System (Bio-Rad).

### Assessment of the presence of a G-quadruplex in segment C

#### Sample preparation

Oligonucleotides for segment C of the *CXXC5* Exon3 with a predicted G-quadruplex forming sequence (SegC-G4), its complementary strand (SegC-Comp), and mutant sequences (Mut1, Mut2, and Mut3) were purchased from Oligomer Biotechnology Inc. (Ankara, Turkey). The Pu_22_-G4 forming sequence of the *VEGF* promoter^80^ and dT_32_ were acquired from IDT. Concentrations of oligonucleotide stock solutions were determined by UV–Vis spectroscopy, using the molar extinction coefficient values obtained by IDT OligoAnalyser Tool. All nucleic acid samples were prepared in 25 mM K-phosphate buffer at pH 7.0 in the presence of 70 mM KCl in Millipore water, where the DNA concentration was 3.0 µM per strand. The nucleic acid samples were heated at 93–95 °C for 5 min and cooled down to room temperature overnight in a water bath to assure the formation of the proper secondary structures. Thioflavin T molecule (ThT) was purchased from Sigma Aldrich (St. Louis, MO, USA). ThT stock solution was prepared in Millipore water, and the concentration of the stock solution was determined by UV–vis spectroscopy using the molar extinction coefficient value of 36,000 M^−1^ cm^−1^ at 412 nm^79^. Igor Pro Software (WaveMetrics, Inc. Portland, OR, USA) was used for data analysis.

#### Circular Dichroism (CD) spectroscopy

For CD spectroscopy, a Circular Dichroism JASCO J-815 spectropolarimeter (JASCO Inc., Easton, MD, USA) equipped with a Peltier-type temperature control system was used. Spectra of all samples for comparison were recorded at 5 °C using 10 mm quartz cells (3.5 mL, 111-QS, Hellma). The CD thermal denaturation experiment for SegC-G4 was performed by varying the temperature from 15 to 95 °C (and reverse) with a 5 °C/min increment and a 1-min waiting period for each temperature point. The Tm value for SegC-G4 was determined by the differentiation of the Normalized Ellipticity (mdeg) at 262 nm vs Temperature (°C) curve.

#### UV–Vis absorption spectroscopy

Cary 8454 spectrophotometer (Agilent Technologies; Santa Clara, CA, USA) equipped with a Peltier-type temperature control system was used for the recording of the UV–Vis absorption spectrum of the samples. UV–Vis thermal denaturation experiments were performed by changing the temperature between 15 and 95 °C (and reverse) with 2 °C/min increments.

#### Fluorescence spectroscopy

Fluorescence spectroscopy was performed by Cary Eclipse Fluorescence Spectrometer (HORIBA Ltd., Kyoto, Japan). All oligonucleotide samples were prepared before experiments with the same annealing procedure described above. Parameters for the fluorescence experiments were: Emission spectra collected between 430 and 700 nm, an excitation wavelength of 412 nm, 5.0 nm excitation and emission slit widths, operation at 800 V and 600 nm/min scan rate.

For the fluorescence experiments, 0.5 µM ThT and 2.0 µM nucleic acid concentrations were selected as the optimal amounts. The results were demonstrated by plotting a bar graph of F − F_0_, where F_0_ is the fluorescence of ThT alone and F is the fluorescence of ThT after the addition of the oligonucleotides at 488 nm.

### Statistical analysis

Experiments were repeated at least two independent times. Results, where and when appropriate, were presented as the mean ± standard error (S.E.) of three biological replicates. Statistical analyses were performed using a two-tailed unpaired t-test with a confidence interval, minimum, of 95%.

## Supplementary Information

Supplementary Information.
