# Supplementary material for: A CpG island promoter drives the CXXC5 gene expression
Source: Sci Rep. 2021 Aug 2;11:15655. doi: 10.1038/s41598-021-95165-6 (PMC8329181; doi:10.1038/s41598-021-95165-6)
Supplement: Supplementary file 1 — Supplementary Information. [file 41598_2021_95165_MOESM1_ESM.pdf]

**Supplementary Information**

# A CpG island promoter drives the CXXC5 gene expression

Pelin Yaşar[1*,7, 8], Gizem Kars[1*], Kerim Yavuz[1*], Gamze Ayaz[1,2], Çerağ Oğuztüzün[3], Ecenaz Bilgen[4], Zeynep Suvacı[4], Özgül Persil Çetinkol[4], Tolga Can[5], Mesut Muyan[1,6,8]

[1]Department of Biological Sciences, Middle East Technical University, Ankara 06800, Turkey; [3]Department of Computer Engineering, Bilkent University, Ankara 06800, Turkey; [4]Department of Chemistry, Middle East Technical University, Ankara 06800, Turkey; [5]Department of Computer Engineering, Middle East Technical University, Ankara 06800, Turkey; [6]Cansyl Laboratories, Middle East Technical University, Ankara 06800, Turkey

[2]Current Address: Cancer and Stem Cell Epigenetics Section, Laboratory of Cancer Biology and Genetics, Center for Cancer Research, National Cancer Institute, National Institutes of Health, Bethesda, MD, 20892, USA

[7]Current Address: Epigenetics and Stem Cell Biology Laboratory, Single Cell Dynamics Group, National Institute of Environmental Health Sciences, Research Triangle Park, NC 27709, USA

[8]Corresponding Authors: pelin.yasar@nih.gov; mmuyan@metu.edu.tr

*Equal contributions

**Short title**: A CpG Island promoter drives the *CXXC5* expression

**Keywords**: CXXC5, CpG Island, Promoter, G-quadruplex, ELF1, MAZ

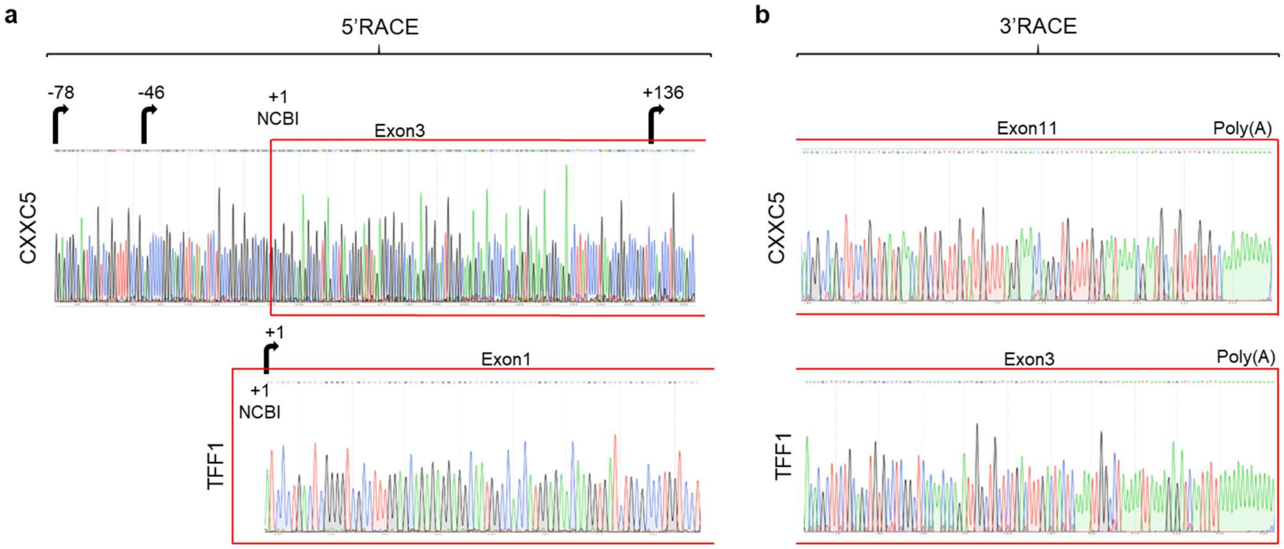


**Figure S1. 5'RACE and 3'RACE**. (**a**) The chromatograms of the longest sequence of *CXXC5*-TV2 and *TFF1* transcripts identified by 5'RACE are shown. Arrows indicate transcription start sites, and +1 denotes the start of the first exon, according to NCBI. (**b**) The chromatographies or chromatograms of the last exon of *CXXC5* and *TFF1* transcripts are also shown. PolyA tails are indicated.

**Exon3 and Surrounding Sequences**

Segment A

-117 agggaggggagccgaggtggagggggttgggggg aggagggagcggcgagtccggtccgggttttgccggcagcccccgggcagcgttcatagctcctgcccg

+1

GgcgggcgcgcggcGGCGGCGGCAGAGGCGGCTGAGCCTGAGCGGGGATGTAGAGGCGGCGGCAGCAGAGGCGG

CACTGGCGGCAAGAGCAGACGCCCGAGCCGAGCGAGAAGAGCGGCAGAGCCTTATCCCCTGAAGCCGGGCCC

Segment B

CGCGTCCCAGCCCTGCCCAGCCCGCGCCCAGCCATGCGCGCCGCCTGCTGAGTCCGGGCGCCGCACGCTGAG

CCCTCCGCCCGCGAGCCGCGCTCAGCTCGGGGGTGATTAGTTGCTTTTTGTTGTTTTTTAATTT*GGGCCGCGGG*

Segment C

*GAGGGGGAGGAGGGG*CAGGTGCTGCAGGCTCCCCCCCCTCCCCGCCTCGGGCCAGCCGCGGCGGCGCGACT

CGGGCTCCGGACCCGGGCACTGCTGGCGGCTGGAGCGGAGCGCACCGCGGCGGTGGTGCCCAGAGCGGAG

Segment D

CGCAGCTCCCTGCCCCGCCCCTCCCCCTCGGCCTCGCGGCGACGGCGGCGGTGGCGGCTTGGACGACTCGG

AGAGCCGgtaggtgtcgggccacggccctccctcgaccccccccggaggccggccccctccccttccc +558

**Figure S2. Exon3 and surrounding DNA sequences.** Segments (A-D) indicate DNA fragments used in luciferase reporter assays to assess the presence of promoter elements of the transcript variant 2 of *CXXC5*. The underlined sequences indicate the overlapping sequences between segments.

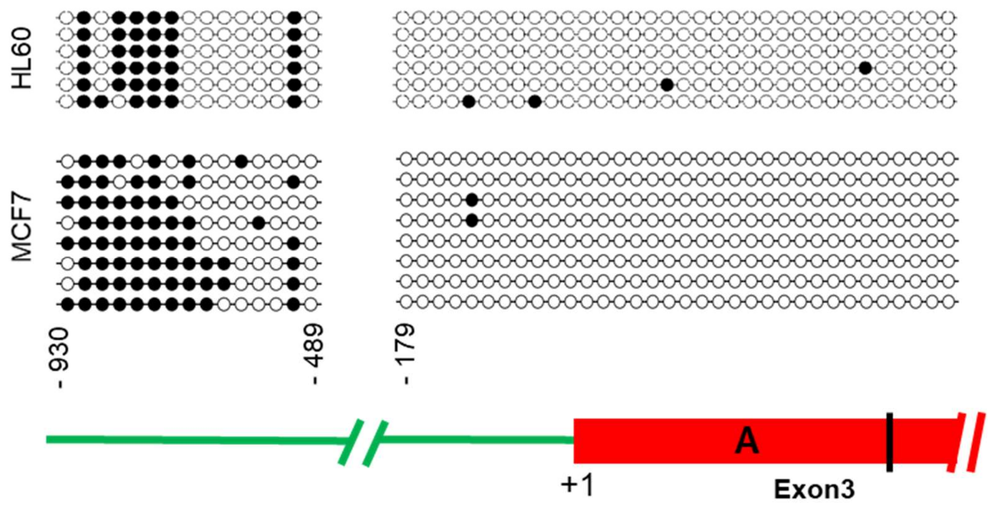


**Figure S3. The methylation state of the *CXXC5* promoter region in HL60 and MCF7 cells.** The methylation of the promoter region (-930 to -489 and -179 through Segment A; +1 indicates the beginning of Exon3) was examined with bisulfite sequencing. Isolated genomic DNA of HL60 or MCF7 cells was subjected to bisulfite reaction for the conversion of unmethylated cytosine residues to uracil followed by bisulfite PCR. PCR amplicons produced with bisulfite primers were cloned and sequenced. Sequence results from six clones from HL60 and eight clones from MCF7 cells derived from two independent experiments are shown. Aligned sequences to the corresponding *CXXC5* regions were depicted as a lollipop distribution. Filled circles indicate methylated and empty circles denote unmethylated CpG dinucleotides.

Gene Expression

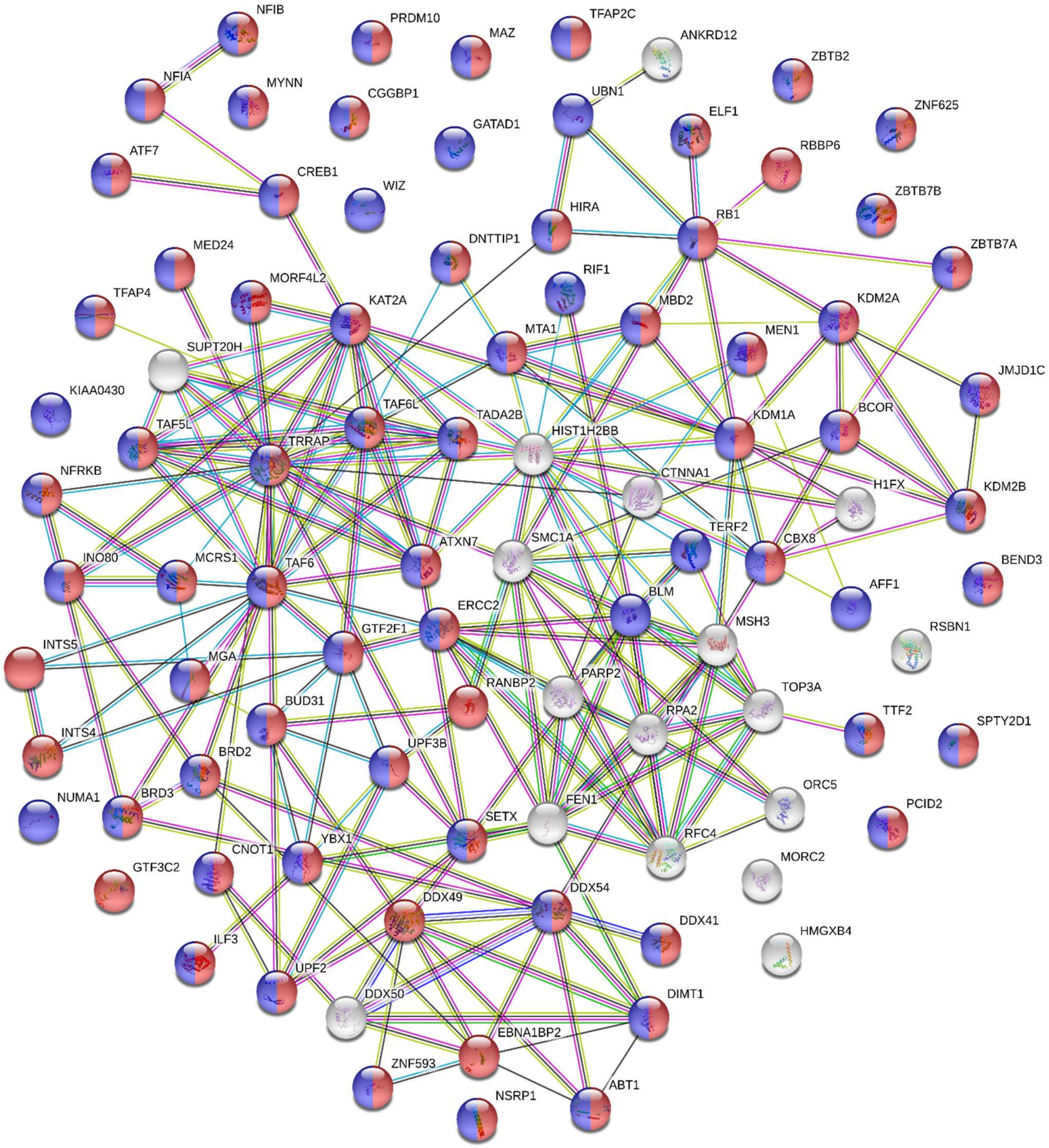


**Figure S4. GO term enrichment analyses of proteins associated with Segment A as the core *CXXC5* promoter element using the STRING interaction database.** Nuclear extracts of MCF7 cells were incubated overnight with a 5'-end biotinylated PCR amplicon containing Segment A or a fragment of Exon10 as the control followed by precipitation with streptavidin-conjugated magnetic beads. Bound proteins were then subjected to MS for protein IDs. Proteins are grouped under the heading of Gene Expression [GO: 0010467 Regulation of gene expression (red) and GO: 0010468, Gene expression (Blue)].

Transcription Factor/Co-Regulator

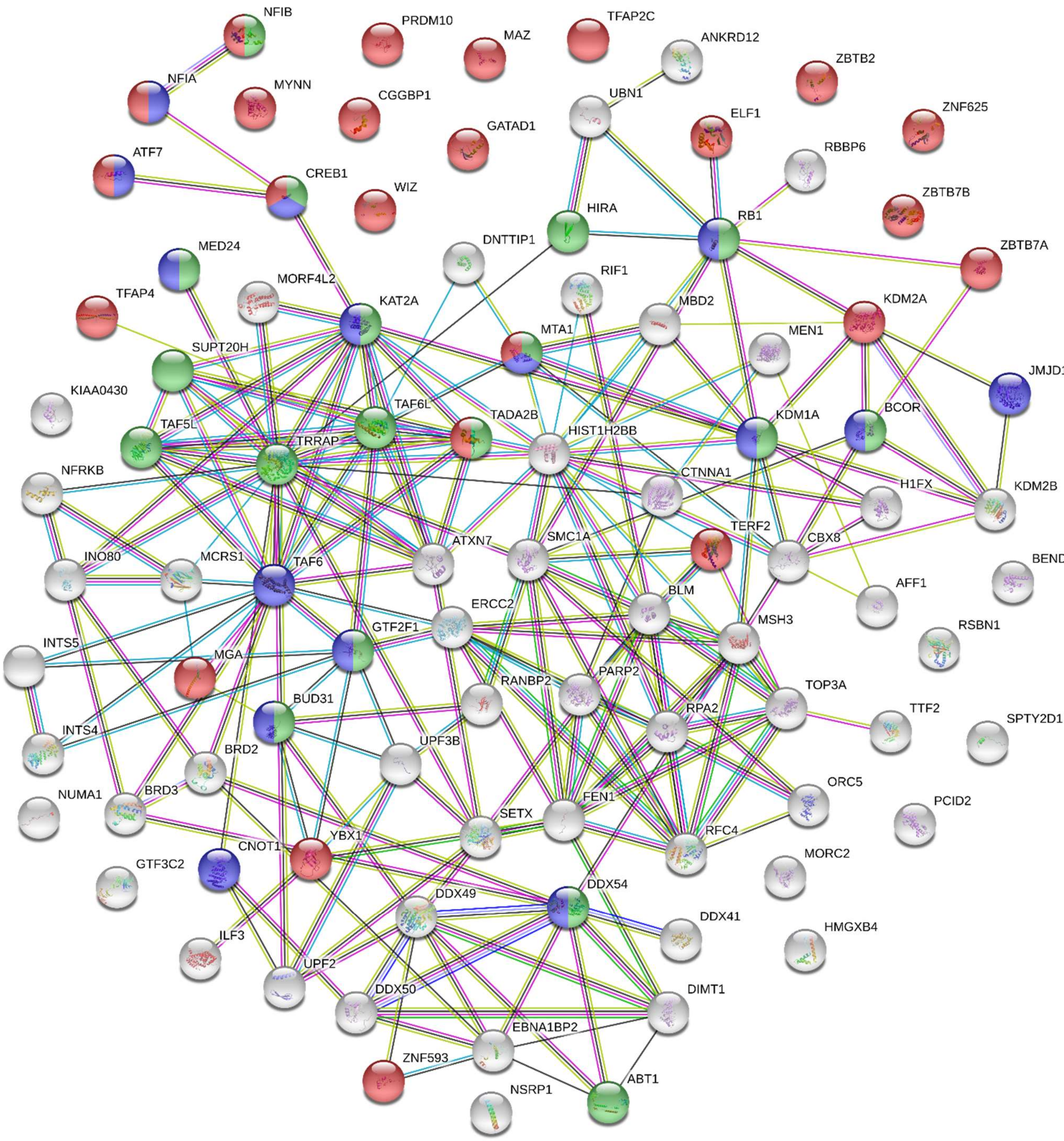


**Figure S5. GO term enrichment analyses of proteins associated with Segment A as the core *CXXC5* promoter element using the STRING interaction database.** Nuclear extracts of MCF7 cells were incubated overnight with a 5'-end biotinylated PCR amplicon containing Segment A or a fragment of Exon10 as the control followed by precipitation with streptavidin-conjugated magnetic beads. Bound proteins were then subjected to MS for protein IDs. Proteins are grouped under the heading of Transcription Factor/Co-Regulator [GO: 0000981, DNA-binding transcription factor activity (red), GO:0008134, Transcription factor binding (blue), and GO: 0003712, Transcription co-regulator activity (green)].

Chromatin & Histones

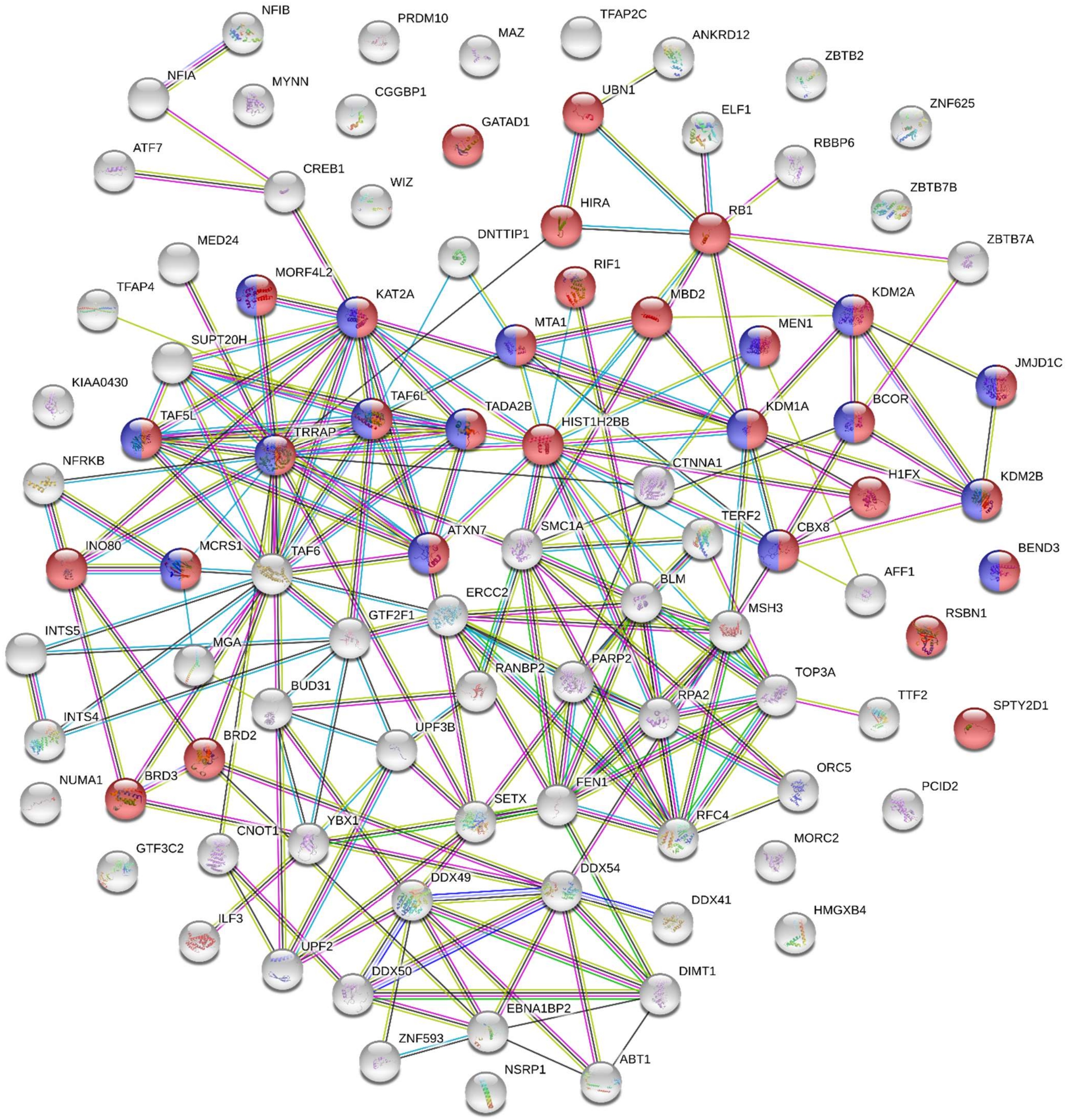


**Figure S6. GO term enrichment analyses of proteins associated with Segment A as the core *CXXC5* promoter element using the STRING interaction database.** Nuclear extracts of MCF7 cells were incubated overnight with a 5'-end biotinylated PCR amplicon containing Segment A or a fragment of Exon10 as the control followed by precipitation with streptavidin-conjugated magnetic beads. Bound proteins were then subjected to MS for protein IDs. Proteins are grouped under the heading of Chromatin and Histone [GO: 0006325, Chromatin organization (red) and GO: 0016570, Histone modification (blue)].

RNA Processing

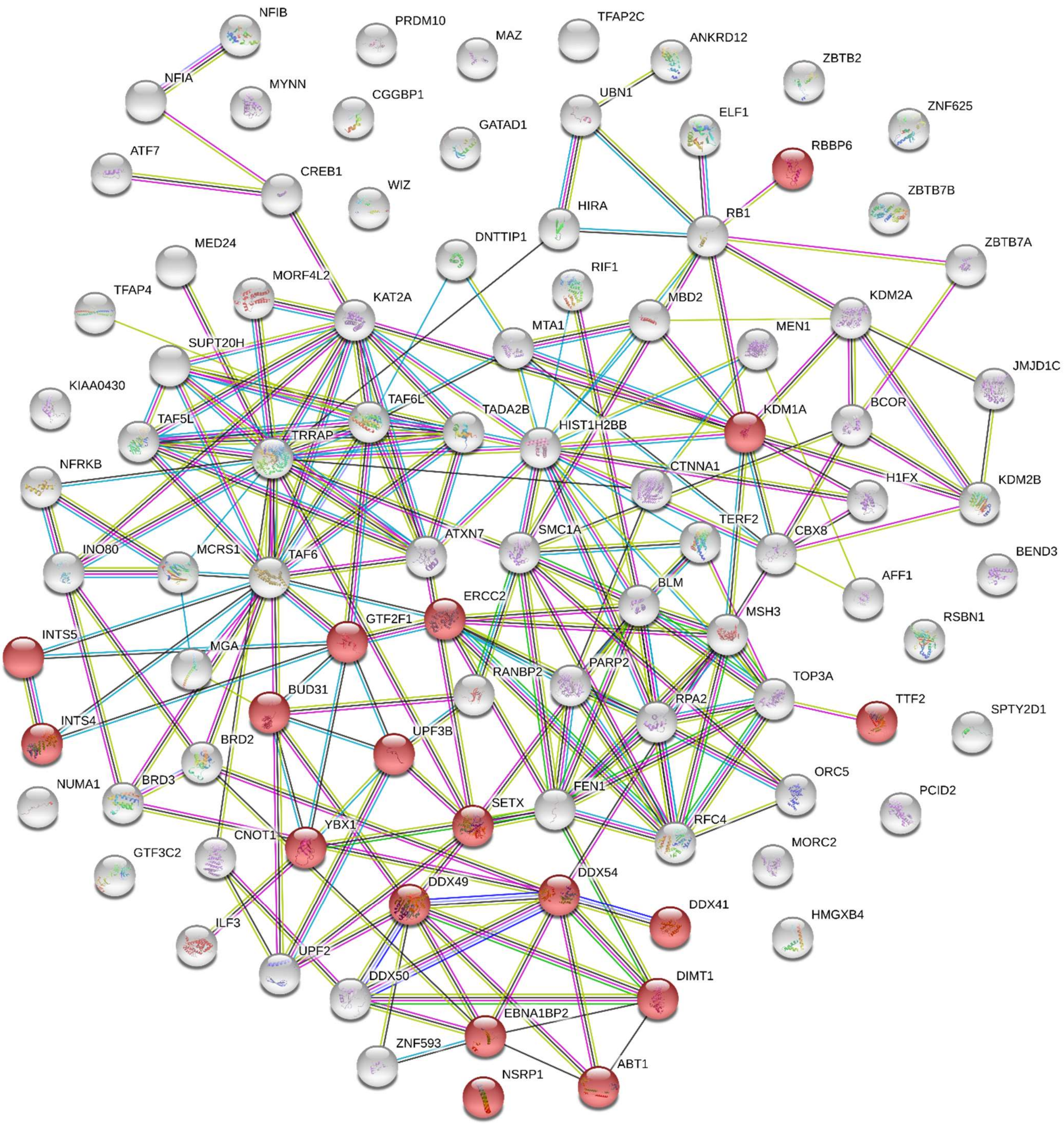


**Figure S7. GO term enrichment analyses of proteins associated with Segment A as the core *CXXC5* promoter element using the STRING interaction database.** Nuclear extracts of MCF7 cells were incubated overnight with a 5'-end biotinylated PCR amplicon containing Segment A or a fragment of Exon10 as the control followed by precipitation with streptavidin-conjugated magnetic beads. Bound proteins were then subjected to MS for protein IDs. Proteins are grouped under the heading of RNA Processing [GO: 0006396, RNA processing (red)].

DNA Modifications

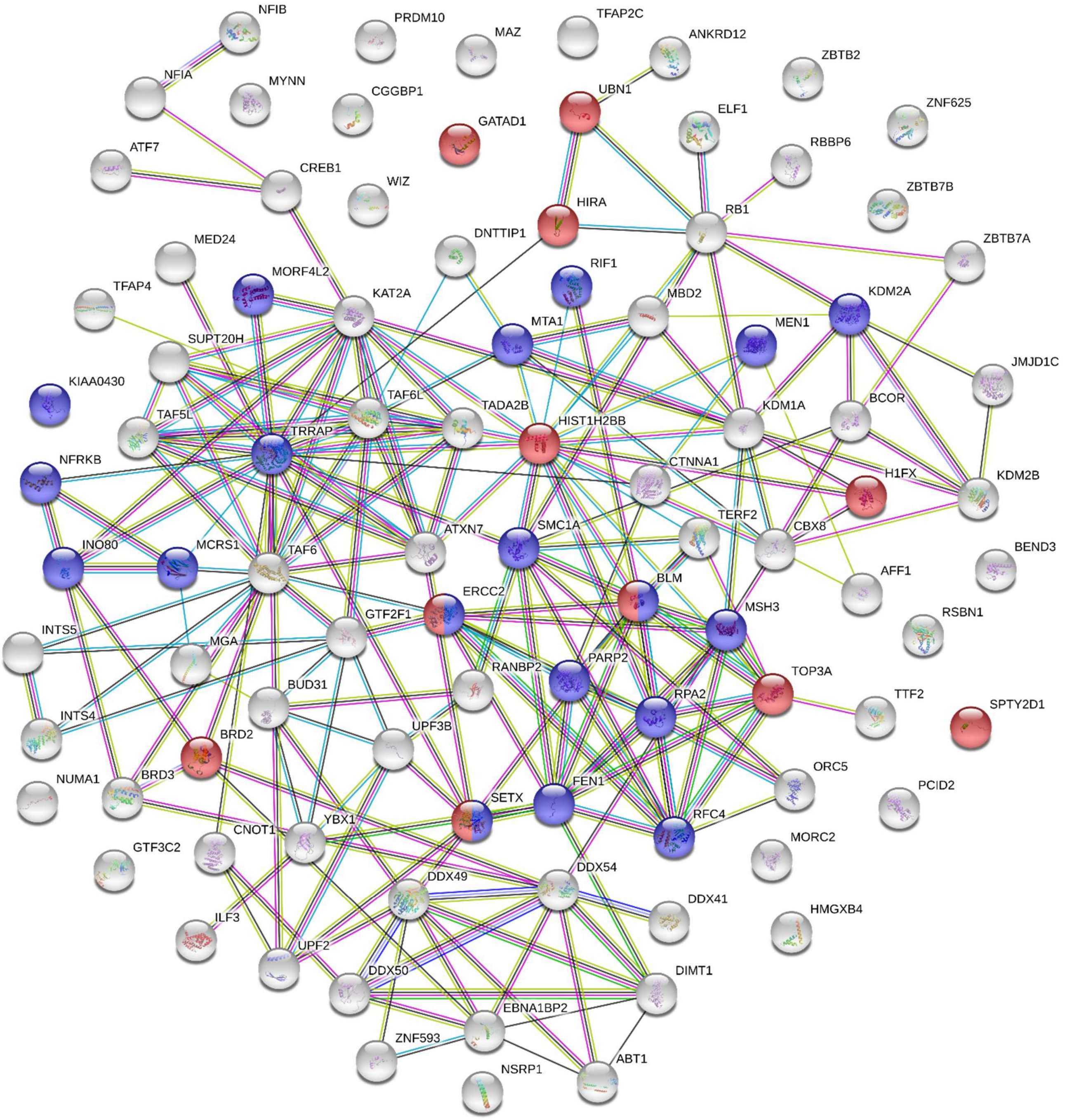


**Figure S8. GO term enrichment analyses of proteins associated with Segment A as the core *CXXC5* promoter element using the STRING interaction database.** Nuclear extracts of MCF7 cells were incubated overnight with a 5'-end biotinylated PCR amplicon containing Segment A or a fragment of Exon10 as the control followed by precipitation with streptavidin-conjugated magnetic beads. Bound proteins were then subjected to MS for protein IDs. Proteins are grouped under the heading of DNA Modifications [GO:0006281, DNA repair (blue) and GO: 0071103, Conformational changes (red)].

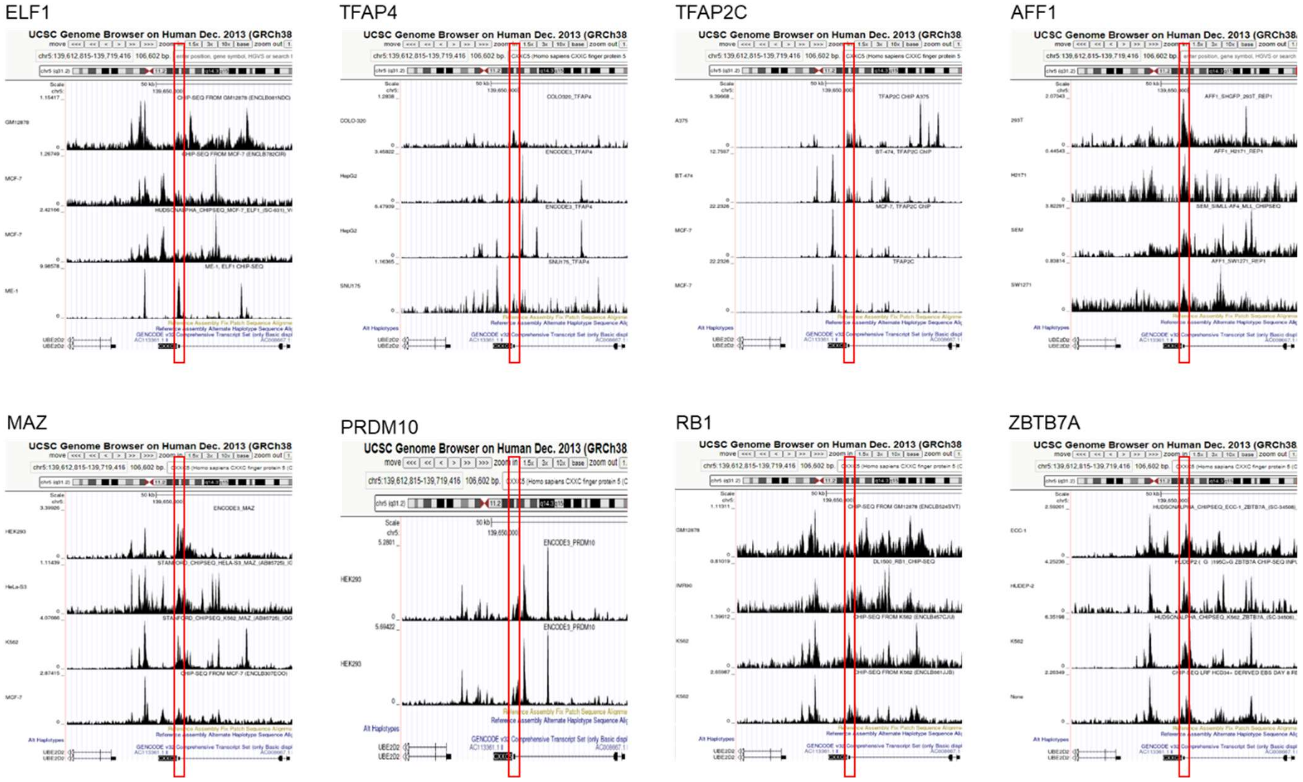


**Figure S9. Binding profiles of transcription factors to the *CXXC5* promoter.** Snapshots of binding profiles generated with Cistrome of various transcription factors identified by the promoter pull-down approach at the CXXC5 gene loci using the UCSC genome browser are shown. The red box indicates Exon3 as the region containing the *CXXC5* promoter.

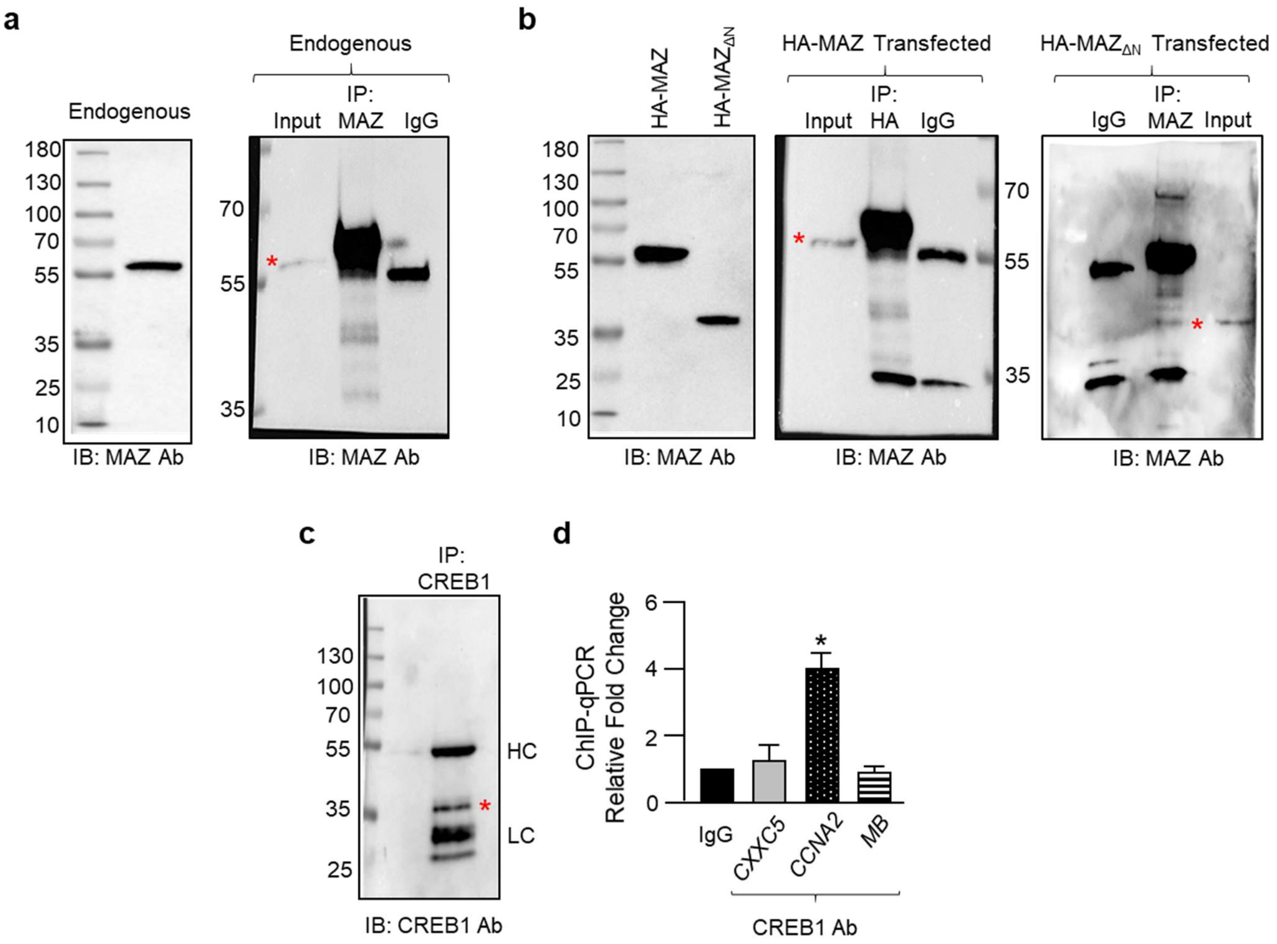


**Figure S10. WB and ChIP-WB.** (**a**) To assess the efficiency of the antibody directed to the carboxyl-terminus of MAZ, to detect and immunoprecipitate MAZ in WB and immunoprecipitations, MCF7 nuclear extracts were subjected to WB analysis. Fragmented chromatin from MCF7 cells processed for ChIP was immunoprecipitated with a species-specific IgG or the MAZ-specific antibody. Samples were then subjected to WB using the MAZ antibody. Input and molecular masses (MM) in kDa are indicated. (**b**) Transiently transfected MCF7 cells with an expression vector bearing the HA-MAZ or the HA-MAZ$_{\Delta N}$ were subjected to WB using the MAZ antibody. Cells were also subjected to ChIP using IgG or the MAZ antibody, followed by WB using the MAZ antibody. Input and MM are indicated. **(c)** Fragmented chromatin from MCF7 cells was processed for ChIP and immunoprecipitated with an antibody specific to CREB1. Asterisk (*) indicates CREB1. **(d)** ChIP from MCF7 cells was carried out with IgG or the CREB1 antibody. Purified DNA was subjected to qPCR with the primer set specific to Segment A or the promoter of *CCNA2* as positive control and Exon2 of *MB* as a negative control. The mean ± SD of three independent experiments is shown—asterisk (*) indicates significant differences depicted as fold change compared to IgG.

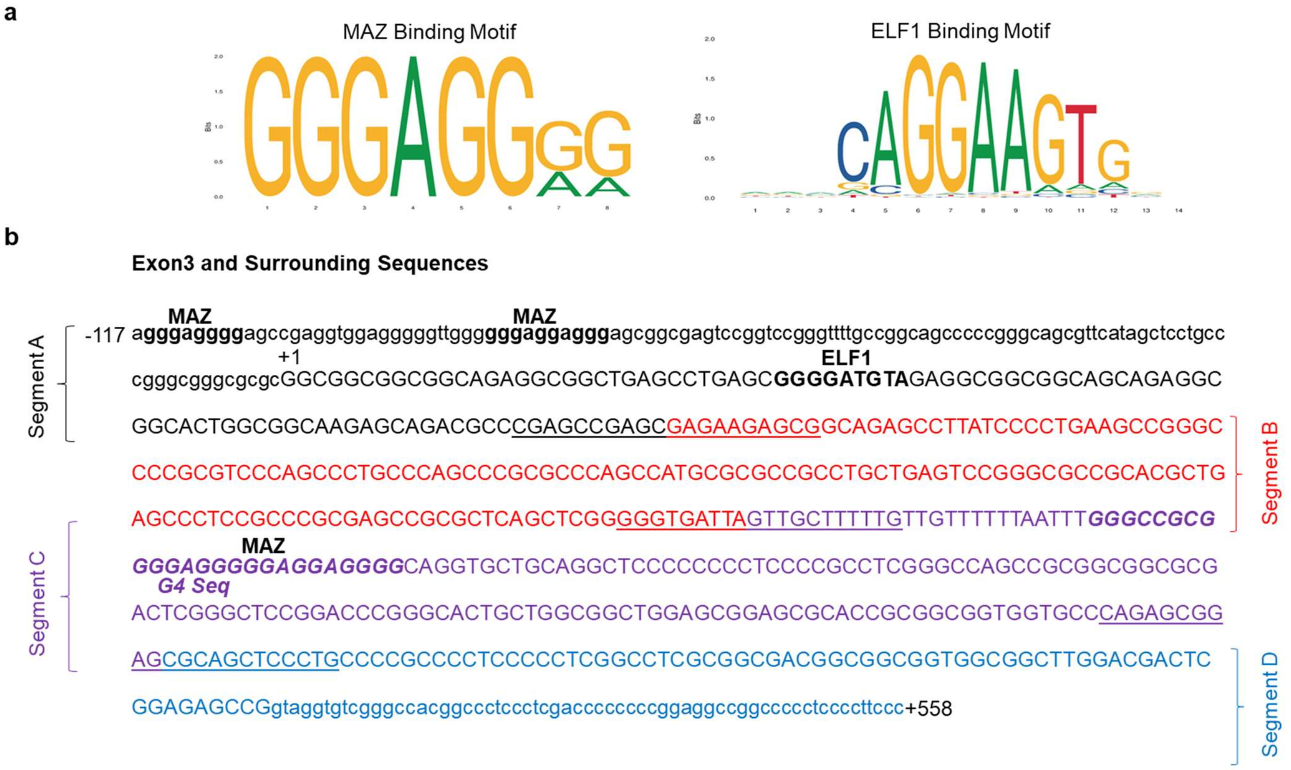


**Figure S11. Binding motifs of ELF1 and MAZ on Exon3 and surrounding sequences.** (**a**) Snapshots of binding motifs generated with JASPAR of ELF1 and MAZ (**b**) Binding sequences for ELF1 and MAZ on Exon3 (bold) and its surrounding sequences are indicated. The possible G-quadruplex forming sequence in Segment C is italicized and bolded. +1 indicates the beginning of Exon3.

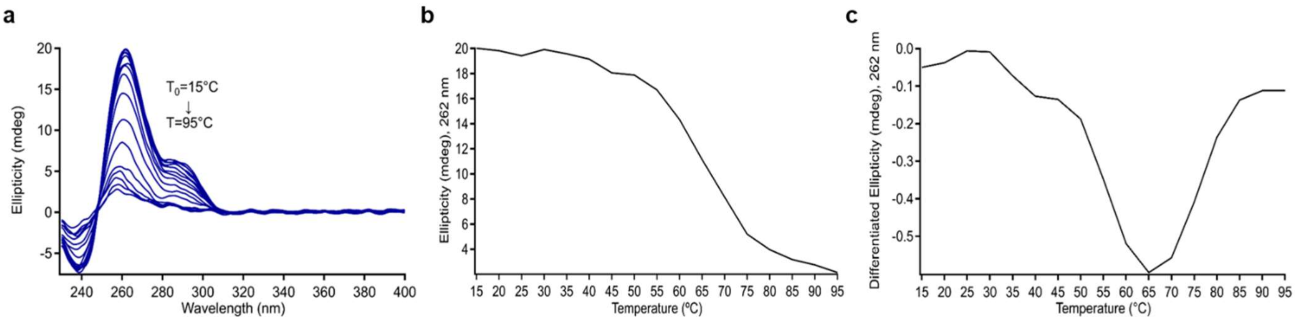


**Figure S12. Thermal denaturation of SegC-G4.** (**a**) CD spectra of SegC-G4 between 15°C and 95°C **(b)** Thermal denaturation profile obtained by monitoring the change in ellipticity at 262 nm with varying temperatures **(c)** Differentiated thermal denaturation profile at 262 nm.

## Table 1. Primer List

### Transcript Variant Detection Primers

| Primer Name | Forward Primer Sequence (5' to 3') (1st Round) | Reverse Primer Sequence (5' to 3') (1st Round) | Forward Primer Sequence (5' to 3') (2nd Round) | Reverse Primer Sequence (5' to 3') (2nd Round) | Forward Primer Sequence (5' to 3') (3nd Round) | Reverse Primer Sequence (5' to 3') (3nd Round) |
|---|---|---|---|---|---|---|
| TV1 | ATGTAGAGGCGGCGGCAGCA | TTGTCTGCTGCTCCTGCCTTT | ATGTAGAGGCGGCGGCAGCA | TTGGGAAGCAGAGGCAGGCAGA | - | - |
| TV2 | ATGTAGAGGCGGCGGCAGCA | ACCACCACTGCTGCCAAAAGA | ATGTAGAGGCGGCGGCAGCA | TTGTCTGCTGCTCCTGCCTTT | ATGTAGAGGCGGCGGCAGCA | CATTGGTGCTGCTGCTGCTA |
| TV3 | TGCAAGTCGGCGGAAAGTTT | TTGTCTGCTGCTCCTGCCTTT | AAGTTTGGCTGCGCGGGTT | CATTGGTGCTGCTGCTGCTA | - | - |
| TV4 | ACTGCGTTTTCTTCGCCTCG | ACCACCACTGCTGCCAAAAGA | GAAGGTCGGCCTTGCACAAA | TTGTCTGCTGCTCCTGCCTTT | - | - |
| TV5 | GGCAGAAAGATAGCAGATTTCCCC | ACCACCACTGCTGCCAAAAGA | GGCAGAAAGATAGCAGATTTCCCC | TTGTCTGCTGCTCCTGCCTTT | - | - |
| TV6 | TGCAAGTCGGCGGAAAGTTT | TTGTCTGCTGCTCCTGCCTTT | GGGCTGATGGAGACAGATGT | CATTGGTGCTGCTGCTGCTA | - | - |
| TV7 | TGCAAGTCGGCGGAAAGTTT | TTGTCTGCTGCTCCTGCCTTT | AAGTTTGGCTGCGCGGGTT | TTGGGAAGCAGAGGCAGG | - | - |
| TV8 | GAGATGGGAACAGCTGAAGG | ACCACCACTGCTGCCAAAAGA | CTCATCCTCGCAGTAGCTGG | TTGTCTGCTGCTCCTGCCTTT | - | - |
| TV9 | GCAGATGCTGTTTCATCGGTC | TTGTCTGCTGCTCCTGCCTTT | GGGCTGATGGAGACAGATGT | TTGGGAAGCAGAGGCAGG | GGGCTGATGGAGACAGATGT | TTGGGAAGCAGAGGCAGG |
| TV10 | GCAGATGCTGTTTCATCGGTC | TTGTCTGCTGCTCCTGCCTTT | GCAGATGCTGTTTCATCGGTC | CATTGGTGCTGCTGCTGCTA | - | - |
| TV11 | ATCTCCAAGTGCCCCAGGTGG | ACCACCACTGCTGCCAAAAGA | CAACCACGGAGAGGTGACA | TTGTCTGCTGCTCCTGCCTTT | CAACCACGGAGAGGTGACA | TTGGGAAGCAGAGGCAGG |
| TV12 | ATCTCCAAGTGCCCCAGGTGG | ACCACCACTGCTGCCAAAAGA | CAACCACGGAGAGGTGACA | TTGTCTGCTGCTCCTGCCTTT | CAACCACGGAGAGGTGACA | CATTGGTGCTGCTGCTGCTA |
| TV13 | ATCTCCAAGTGCCCCAGGTGG | ACCACCACTGCTGCCAAAAGA | CAACCACGGAGAGGTGACA | TTGTCTGCTGCTCCTGCCTTT | CAACCACGGAGAGGTGACA | TTGGGAAGCAGAGGCAGG |
| TV14 | ATCTCCAAGTGCCCCAGGTGG | ACCACCACTGCTGCCAAAAGA | ATCTCCAAGTGCCCCAGGTGG | TTGTCTGCTGCTCCTGCCTTT | ATCTCCAAGTGCCCCAGGTGG | TTGGGAAGCAGAGGCAGG |

### qPCR Primers for Transcript Variants

| Primer Name | Forward Primer Sequence (5' to 3') | Reverse Primer Sequence (5' to 3') |
|---|---|---|
| TV1 | CAGCTCGGGGGTGATTAGTTG | ATCGCTTGAGCTCAGGAGTTT |
| TV2 | CAGCTCGGGGGTGATTAGTTG | AACAGAGATGCCTGGTGGGCCT |
| TV3 | AAGTTTGGCTGCGCGGGTT | AACAGAGATGCCTGGTGGGCCT |
| TV4 | ACTGCGTTTTCTTCGCCTCG | TCCCAACTGCATATAATCCCA |
| TV5 | CGCGGCCAAATTCTCCCTCCC | CTCCGGGGAAATCTGCTATCT |
| TV6 | TGCAAGTCGGCGGAAAGTTT | ACATCTGTCTCCATCAGCCC |
| TV7 | TGCAAGTCGGCGGAAAGTTT | ATCGCTTGAGCTCAGGAGTTT |
| TV8 | AACAGCTGAAGGAGATTGGCC | CCAGCTACTGCGAGGATGAGG |
| TV10 | AGCCTTCCCCAGGGGCCTTGT | AACAGAGATGCCTGGTGGGCCT |

### Cloning Primers for *Firefly* Luciferase Vector

| Primer Name | Forward Primer Sequence (5' to 3') | Reverse Primer Sequence (5' to 3') |
|---|---|---|
| Full Length Region | ATCTCAGGCTGTAACGTTTAACC | TGTCTGAACTCAGTGATCTGC |
| Truncated 1 | CAGTACCTATTGAGCGCTGC | TGTCTGAACTCAGTGATCTGC |
| Truncated 2 | ACCCTCGACTAGGAATCTCT | TGTCTGAACTCAGTGATCTGC |
| Truncated 3 | AGGGAGGGGAGCCGAGGTGGA | TGTCTGAACTCAGTGATCTGC |
| Truncated 4 | AGGGAGGGGAGCCGAGGTGGA | GGGAAGGGGAGGGGGCCGGCC |
| Truncated 5 | GGCCGGCCCCCTCCCCTTCCC | TGTCTGAACTCAGTGATCTGC |
| Segments A-B-C-D | AGGGAGGGGAGCCGAGGTGGA | GGGAAGGGGAGGGGGCCGGCC |
| Segments A-B-C | AGGGAGGGGAGCCGAGGTGGA | CAGGGAGCTGCGCTCCGCTCTG |
| Segments B-C-D | CGAGCCGAGCGAGAAGAGCG | GGGAAGGGGAGGGGGCCGGCC |
| Segments A-B | AGGGAGGGGAGCCGAGGTGGA | CAAAAAGCAACTAATCACCC |
| Segments B-C | CGAGCCGAGCGAGAAGAGCG | CAGGGAGCTGCGCTCCGCTCTG |
| Segments C-D | GGGTGATTAGTTGCTTTTTG | GGGAAGGGGAGGGGGCCGGCC |
| Segment A | AGGGAGGGGAGCCGAGGTGGA | CGCTCTTCTCGCTCGGCTCG |
| Segment B | CGAGCCGAGCGAGAAGAGCG | CAAAAAGCAACTAATCACCC |
| Segment C | GGGTGATTAGTTGCTTTTTG | CAGGGAGCTGCGCTCCGCTCTG |
| Segment D | CAGAGCGGAGCGCAGCTCCCTG | GGGAAGGGGAGGGGGCCGGCC |

### Bisulfite Conversion and Sequencing Primers

| Primer Name | Forward Primer Sequence (5' to 3') | Reverse Primer Sequence (5' to 3') |
|---|---|---|
| First Region | ATTTTAAAGAATTGGAGAAAATTTTTATTT | AATTCCTAATCAAAAATCCTAAAAAATAAA |
| Second Region | TTATTTTTTAGGATTTTTGATTAGGAATTTT | CTTCAAAAAATAAAACTCTACC |
| Third Region | TTTGAGTTGAGTGAGAAGAGTGG | AATCACCCCCAAACTAAACACA |
| Fourth Region | TTTTTTTTGTTTCGGGTTAGTTGTG | CACCTACCAACTCTCCAAATCA |
| Fifth Region | GTGTGTTTATAGATTGGTAGTTTTTTTA | CTCGAAAAATAATATATCATCT |

### Mnase Assay Primers

| Primer Name | Forward Primer Sequence (5' to 3') | Reverse Primer Sequence (5' to 3') |
|---|---|---|
| T1 Region | ACCCTCGACTAGGAATCTCT | TCCACCTCGGCTCCCCTCCC |
| T2 Region | GCTGCGCTGGGAGGGAGGCGG | ACTAATCACCCCCGAGCTGAG |
| T3 Region | ATGTAGAGGCGGCGGCAGCA | TCCCCGCGGCCCAAATTAAAA |
| T4 Region | AGAGCCTTATCCCCTGAAGC | GCTCTCCGAGTCGTCCAA |
| T5 Region | ACTCGGAGAGCCGGTAGGTGT | GACCGAGCATACTGCGGCTTA |
| M1 Region | GCTGCGCTGGGAGGGAGGCGG | CGGGCAGGAGCTATGAAC |
| M2 Region | AGAGCCTTATCCCCTGAAGC | ACTAATCACCCCCGAGCTG |
| M3 Region | GCACTGCTGGCGGCTGGA | ACACCTACCGGCTCTCCGAGT |

### Histone ChIP Primers

| Primer Name | Forward Primer Sequence (5' to 3') | Reverse Primer Sequence (5' to 3') |
|---|---|---|
| Segment A | CGAGGTGGAGGGGGTTG | GCAGGAGCTATGAACGCTGC |
| Segment B | CTGAGCGGGGATGTAGAGG | GCTTCAGGGGATAAGGCTCTGC |
| Segment C | AGAGCCTTATCCCCTGAAGC | ACTAATCACCCCCGAGCTG |
| Segment D | GCACTGCTGGCGGCTGGA | GCTCTCCGAGTCGTCCAA |
| GAPDH Promoter | GAATGGGCAGCCGTTAGGAA | GCATCACCCGGAGGAGAAAT |

### ChIP Primers

| Primer Name | Forward Primer Sequence (5' to 3') | Reverse Primer Sequence (5' to 3') |
|---|---|---|
| CXXC5 Promoter | AGGGAGGGGAGCCGAGGTGGA | CGGGCAGGAGCTATGAAC |
| OAS1 Promoter | AATTCAGCACTGGGATCAGG | TTGGCTGGGGTATTTCTGAG |
| C-MYC Promoter | CTTTATAATGCGAGGGTCTGG | TGCCTCTCGCTGGAATTACT |
| CCNA2 Promoter | ATCCCGCGACTATTGAAATG | CGCTCACTAGGTGGCTCAG |
| MB Exon2 | AAGTTTGACAAGTTCAAGCACCTG | TGGCACCATGCTTCTTTAAGTC |
| GAPDH Promoter | TACTAGCGGTTTTACGGGCG | TCGAACAGGAGGAGCAGAGAGCGA |

### 5'RACE Primers and Adapter Sequences

| | Adapter Sequence (5' to 3') |
|---|---|
| **5'RACE Adapter** | GCUGAUGGCGAUGAAUGAACACUGCGUUUGCUGGCUUUGAUGAAA |

| Primer Name | Forward Primer Sequence (5' to 3') | Reverse Primer Sequence (5' to 3') |
|---|---|---|
| Kit Outer | GCTGATGGCGATGAATGAACACTG | |
| Kit Inner | CGCGGATCCGAACACTGCGTTTGCTGGCTTTGATG | |
| CXXC5 Outer | | GCTCTCCGAGTCGTCCAA |
| CXXC5 Inner | | ACTAATCACCCCCGAGCTG |
| TFF1 Outer | | TTCACACTCCTCTTCTGGAGGG |
| TFF1 Inner | | TATTTGCACACTGGGAGGGCGT |

### 3'RACE Primers and Adapter Sequences

| | Adapter Sequence (5' to 3') |
|---|---|
| **3'RACE Adapter** | GCGAGCACAGAATTAATACGACTCACTATAGGT12VN |

| Primer Name | Forward Primer Sequence (5' to 3') | Reverse Primer Sequence (5' to 3') |
|---|---|---|
| 3'RACE Adapter | GCGAGCACAGAATTAATACGACTCACTATAGGT12VN | |
| Kit Outer | | GCGAGCACAGAATTAATACGACT |
| Kit Inner | | CGCGGATCCGAATTAATACGACTCACTATAGG |
| CXXC5 Outer | TAGAAGCCGCTCTGTATCCATCCA | |
| CXXC5 Inner | CCTTGCTAATGGCAACAGAAGA | |
| TFF1 Outer | GTCCCTCCAGAAGAGGAGTGT | |
| TFF1 Inner | TTCTGCAGGGATCTGCCTGCAT | |

### Northern Blot Primers

| Primer Name | Forward Primer Sequence (5' to 3') | Reverse Primer Sequence (5' to 3') |
|---|---|---|
| Vector Primers | 5'BiosgTAGCAAAATAGGCTGTCCCC | TGGCTTTACCAACAGTACCGGAAT |
| Cloning of Exon3 Probe Sequence | AGAGCCTTATCCCCTGAAGC | GCTCTCCGAGTCGTCCAA |
| Cloning of Exon10-11 Probe Sequence | TGAGTACCCCATGCAG | GACGGGTGCATCCGTT |
| Gapdh | TCACCACCATGGAGAA | CCAGCGTCAAAGGTGG |

**Table 2. Proteins Associated with Segment A**

| Protein | | Protein | | Protein | | Protein |
|---|---|---|---|---|---|---|
| | | | | | | |
| ABT1 | | FEN1 | | MORF4L2 | | TAF5L |
| AFF1 | | GATAD1 | | MSH3 | | TAF6 |
| ANKRD12 | | GTF2F1 | | MTA1 | | TAF6L |
| ATF7 | | GTF3C2 | | MYNN | | TERF2 |
| ATXN7 | | H1FX | | NFIA | | TFAP2C |
| BCOR | | HIRA | | NFIB | | TFAP4 |
| BEND3 | | HIST1H2BB | | NFRKB | | TOP3A |
| BLM | | HMGXB4 | | NSRP1 | | TRRAP |
| BRD2 | | ILF3 | | NUMA1 | | TTF2 |
| BRD3 | | INO80 | | ORC5 | | UBN1 |
| BUD31 | | INTS4 | | PARP2 | | UPF2 |
| CBX8 | | INTS5 | | PCID2 | | UPF3B |
| CGGBP1 | | JMJD1C | | PRDM10 | | WIZ |
| CNOT1 | | KAT2A | | RANBP2 | | YBX1 |
| CREB1 | | KDM1A | | RB1 | | ZBTB2 |
| CTNNA1 | | KDM2A | | RBBP6 | | ZBTB7A |
| DDX41 | | KDM2B | | RFC4 | | ZBTB7B |
| DDX49 | | MARF1 | | RIF1 | | ZNF593 |
| DDX50 | | MAZ | | RPA2 | | ZNF625 |
| DDX54 | | MBD2 | | RSBN1 | | |
| DIMT1 | | MCRS1 | | SETX | | |
| DNTTIP1 | | MED24 | | SMC1A | | |
| EBNA1BP2 | | MEN1 | | SPTY2D1 | | |
| ELF1 | | MGA | | SUPT20H | | |
| ERCC2 | | MORC2 | | TADA2B | | |
